# Natural attenuation processes control groundwater contamination in the Chernobyl exclusion zone: evidence from 35 years of radiological monitoring

**DOI:** 10.1038/s41598-022-22842-5

**Published:** 2022-10-29

**Authors:** Dmitri Bugai, Sergey Kireev, Mohammad A. Hoque, Yuri Kubko, Jim Smith

**Affiliations:** 1 Institute of Geological Sciences, Kyiv, Ukraine; 2 State Special Enterprise “Ecocenter”, Chernobyl, Ukraine; 3 University of Portsmouth, Portsmouth, UK

**Keywords:** Environmental impact, Hydrology, Environmental sciences

## Abstract

The Chernobyl Exclusion Zone (CEZ) contains the vast majority of radionuclides released by the accident in nuclear fuel particle form. We present and analyze groundwater measurements collected from the monitoring network in CEZ covering key aquifers over 35 years since the accident. These new data, together with a comprehensive analysis of historical data shows that ^90^Sr remains mobile in the subsurface environment, while groundwater concentrations of ^137^Cs, Pu isotopes and ^241^Am are relatively low, and are not of radiological concern. During the last two decades, ^90^Sr and ^137^Cs levels have declined or remained stable over time in the majority of monitoring locations. This is due to natural attenuation driven by gradual exhaustion of the fuel particle source, geochemical evolution of groundwater downstream from waste dumps and radionuclide retention in surface soil due to absorption and bio-cycling. Decommissioning of the cooling pond and construction of the ‘New safe confinement’ over Unit 4 (damaged reactor) also favored better protection of groundwater close to the Chernobyl plant site. Data from confined and unconfined aquifers, as well as rivers, evidence low radiological risks from groundwater contamination both outside the CEZ and to onsite “self-settlers”. Though several groundwater contamination “hot spots” remain in the vicinity of Unit 4, “Red Forest” waste trenches and surface water bodies with contaminated bottom sediments, the findings of this study support a monitored natural attenuation approach to groundwater management in the CEZ.

## Introduction

**Figure 1:**
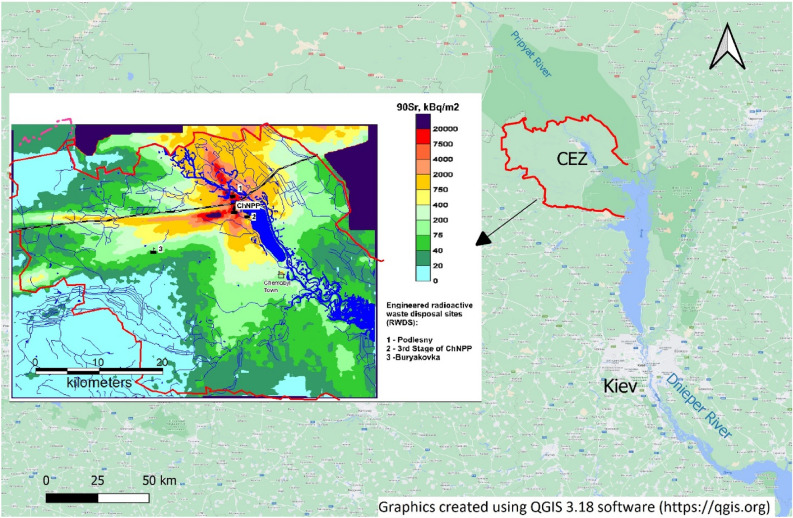
Location of the Chernobyl Exclusion zone (CEZ). Surface contamination map by ^90^Sr in the CEZ (as of 1997) is based on data of^35^.

The Chernobyl accident on 26 April 1986 resulted in the release of radioactivity from the damaged Unit 4 to the atmosphere in the form of aerosols and dispersed nuclear fuel particles (FP) resulting in deposition to the surrounding areas. The highest levels of contamination of environmental media (soils, forests, water bodies) by radioactive fallout containing long-lived radionuclides (^137^Cs, ^90^Sr, ^241^Am and Pu isotopes) were observed in the 30-km zone surrounding the Chernobyl nuclear power plant (ChNPP)^28,35,70^ (Fig. 1). Shortly after the accident the population was evacuated from the 30-km zone (Chernobyl exclusion zone—CEZ) and access to it has remained restricted until the present time.

Activity ratios of ^90^Sr and trans-uranium elements (TUE—Pu isotopes and ^241^Am) in fuel fallout in the CEZ were rather stable (^238^Pu/^90^Sr = 0.0089, ^239+240^Pu/^90^Sr = 0.019, ^241^Pu/^90^Sr = 0.57, ^241^Am/^90^Sr = 0.019 as in 2000), and corresponded to those in the irradiated fuel of Unit 4. The ^137^Cs to ^90^Sr ratio in soils was determined by superposition of fuel and condensation fallout in CEZ. In the near zone (2–5 km distance from ChNPP) where contamination is dominated by FP, this ratio is in the range from 1 to 2^2,35^.

At the time of deposition, radionuclides associated with the FP component were characterized by low mobility in the environment. With time, however, an increase of content of mobile forms of radionuclides (water soluble, ion-exchangeable) has occurred in soils due to chemical weathering (dissolution) of FP under the influence of environmental factors^36,37,41^. The dissolution rate of FP is determined by the characteristics of particles (type of matrix, oxidation state of uranium, presence of their surface of inert materials such as zirconium) and is affected by environmental conditions, in particular by soil solution pH (increasing with acidity) and presence of oxygen^36,37^. In the western trace of radioactive fallout (formed predominantly by non-oxidized FP) the dissolution half-life of FP has been estimated, depending on soil pH, to range from 5 to 14 years, while in the northern and southern traces (formed predominantly by oxidized FP) the dissolution half-life of FP is estimated to be from 1 to 14 years. In acidic soils in the CEZ, FP had already dissolved within 15–20 years following the accident^36^. Within the western trace (which is characterized by relatively high contribution of non-oxidized and Zr-containing FP) about 55% of activity of ^90^Sr and TUE were still associated with FP in Trench no.22 of “Red Forest” site in 2015^37^.

The geomorphology of the CEZ potentially promotes transfer of radionuclides to the groundwater system: the land is mostly flat and surface soils (most often sod-podzols (Podzoluvisol)) are generally sandy with low sorption capacity and relatively high atmospheric water infiltration rates (typically 200–300 mm y^−1^). Three main aquifers are present (from top to bottom): (1) the unconfined aquifer in the Quaternary sandy deposits, (2) the confined aquifer in the Eocene sandy deposits, and (3) the confined aquifer in the Cretaceous chalk deposits^21^. The first two aquifers represent the zone of most intensive ‘atmospheric water—groundwater—surface water‘ exchange (see Supplemental Information, Sect. 1 for more detail on site geology and hydrogeology).

The ChNPP is situated in the middle reaches of the Pripyat River which, as part of the Dnieper river-reservoir system, provides drinking and irrigation water to about 30 million people including Kyiv city 140 km south of the ChNPP. The groundwater (both the shallow phreatic and deep confined aquifers) is used as the main drinking water resource for the local population in the Ukrainian Polessye region, and in particular “self-settlers” in the CEZ.

Potential sources of groundwater contamination in CEZ include ‘diffuse pollution sources’ (topsoil in 30-km zone contaminated by radioactive fallout) and ‘point sources’ including highly contaminated soil in the direct vicinity of the Unit 4 ‘Sarcophagus’, cooling reservoir (“cooling pond”), radioactive waste dumps and engineered radioactive waste storage facilities. The highest levels of topsoil contamination are observed in the 10-km zone of the ChNPP, while surrounding peripheral areas of CEZ often have several orders of magnitude lower levels. About 4% (75 km^2^) in the direct vicinity of the ChNPP has surface contamination densities by ^90^Sr in the range 2000–20,000 kBq m^−2^, about 19% (380 km^2^) – 200 – 2000 kBq m^-2^, and about 77% (1540 km^2^) – 20 – 200 kBq m^-2^ or less (as of 1997)^35^ (Fig. 1). The major activity “hot spot” in the 10-km zone is the so called “local zone “ of the “Sarcophagus”’ (within an approximate100—250 m radius of the facility). Here the radioactivity is concentrated within the ‘accident related’ layer of soil that is ~ 10–30 cm thick, and is covered by an up to 10 m thick ‘post-accident man-made’ layer of sand and concrete. The activity concentration of ^137^Cs in soil in the ‘accident related’ layer is 7.1 10^5^- 1.9 10^9^ Bq kg^-1^, for ^90^Sr – 6.4 10^5^ to 2 10^9^, and for ^239+240^Pu – 1.7 10^3^ to 3 10^7^ Bq kg^-1^ (as of 1998)^56^.

A large-scale redistribution of radioactivity in the 10-km zone occurred in 1986–1988 during site clean-up activities, which consisted in the bulldozing and in-situ burial of the contaminated topsoil layer (~ 20 cm), contaminated vegetation (“Red Forest”), buildings etc. in the 2–3 m deep trenches and above-ground mounds. The decontamination area around the ChNPP encompassed approximately 10 km^2^, the total number of waste burials is estimated at 800–1000, and the volume of buried radioactive materials is estimated at 1.2 million m^3^^52^. The specific activity of buried waste materials in the most contaminated waste burial reach for ^137^Cs – 2.2 10^6^ Bq kg^-1^, for ^90^Sr – 3.3 10^5^ Bq kg^−1^ (as of 2015)^17^. One other important ‘point source’ is the cooling pond, which was contaminated due to releases of highly contaminated water from the Unit 4 emergency cooling system, as well as due radioactive fallout on the water surface. Inventories of key sources of contamination are summarized in Table 1.

**Table 1:** Inventory of total activity of main radiologically important radionuclides (^137^Cs, ^90^Sr, ^238-241^Pu, ^241^Am) in various locations inside the CEZ (activity values decay-corrected to reference date 2015).

Location	Activity inventory, Bq					Reference
	^137^Cs	^90^Sr	^238^Pu	^239+240^Pu	^241^Am	
Radionuclide half-life, years	30.2	28.8	87.7	2.4 10^4^(^239^Pu) 6.5 10^3^(^240^Pu)	432.2	
*Diffuse source*						
*Soil in the Ukrainan part of the CEZ*						
Activity inventory	2 10^15^	5.4 10^14^	6.2 10^12^	1.5 10^13^	2.7 10^13^	^35^
*Point sources*						
Contaminated soil in the local zone of the “Sarcophagus”	1.2 10^15^	1.2 10^15^	9.0 10^12^	2.2 10^13^	4.0 10^13^	^56^
Radioactive waste dump sites *	1.6 10^14^	1.3 10^14^	1.2 10^12^	2.9 10^12^	5.3 10^12^	^52^
Cooling pond (bottom sediments)	1.2 10^14^	1.7 10^13^	2.1 10^11^	5.2 10^11^	9.5 10^11^	^8^

*estimates for individual radionuclides derived from total activity assuming that activity of waste is dominated by the fuel component.

During the first months after the accident, large scale engineering measures were undertaken to mitigate the off-site migration of radionuclides through the groundwater pathway from the CEZ, which included construction of ‘drainage curtains’ composed of hundreds of pumping wells, and a ‘slurry wall’ around the ChNPP. These hastily planned measures were based on “worst case” assumptions regarding contaminant inventory and mobility, and already by the end of 1986 were considered disproportionate and unnecessary^9,11,27^. However, since 1989, increased mobility of ^90^Sr was observed in the CEZ due to gradual dissolution and release of radionuclides from nuclear fuel particles, and groundwater contamination was once again considered a high priority concern. The management approaches for groundwater contamination in following decades were based on more careful monitoring studies and risk assessment analyses^10,52,64^.

The last decade has seen implementation of major projects at the ChNPP site, namely decommissioning of the cooling pond (beginning 2014) and completion of the ‘New safe confinement’ (NSC) above Unit 4 (in 2016), which, among other objectives, were aimed at mitigation of radionuclide migration to the groundwater system. Current planning of decommissioning of the ChNPP, transformation of the Unit 4 to an environmentally safe system and related waste management operations assumes long-term (up to 500 years) institutional control over the inner 10-km zone of ChNPP (“industrial zone”), which contains the main contaminated objects as well as waste storage and disposal facilities. The “industrial zone” is not assumed to be suitable for residential living and producing foodstuffs for at least the next 200 years due to radiological risks caused by external irradiation, inhalation and ingestion exposure pathways^52^. The very long-term (> 100's of years) radiological risks in the 10-km zone are dominated by TUE^47^.

The radiologically important long-lived radionuclides of concern covered by the extensive long-term groundwater monitoring program in CEZ included ^90^Sr, ^137^Cs, Pu isotopes (Pu^238^, Pu^239^, Pu^240^), and ^241^Am. In the light of extensive data published in full for the first time here, we evaluate and discuss processes controlling transport and fate of radionuclides in the subsurface environment of CEZ. We also analyze and discuss on-site and off-site risks, and draw conclusions from 35 years of monitoring studies for managing contaminated groundwater in the future.

## Results and discussion

### Radionuclide migration in soils

Post-accident observations have shown that vertical migration in soil of fallout radionuclides in Chernobyl contaminated areas was a rather slow process. The ability of radionuclides to migrate in soil decreases in the following sequence: ^90^Sr > ^137^Cs > Pu≈^241^Am isotopes^33,34^. For radionuclides initially associated with FP, such as ^90^Sr, radionuclide mobility in soils was limited by dissolution of fuel matrix and radionuclide release to mobile forms^41,43,44^. Studies carried out during the first decade following the accident reported downward migration rates of radionuclides in soil of an order of 1 ± 0.5 cm y^−1^ or less^3,23,25,59^. The downward migration rate of ^90^Sr was typically by 20–50% higher than that of ^137^Cs.

As the second and third decades have passed since the time of Chernobyl accident, more distinct differences were observed in vertical distribution of ^90^Sr and ^137^Cs in soils of CEZ. The ^137^Cs migration in soil has slowed down and reached “quasi-equilibrium” state. The distribution of ^137^Cs in soil profiles typically followed exponential profile with maximum activity still in the 5–10 cm upper layer of soil^28,34,61^. On a whole, similar behavior of ^137^Cs is observed in forest soils affected by Fukushima accident, where its distribution in soil by about the 8th year has stabilized^32,54^.

**Figure 2:**
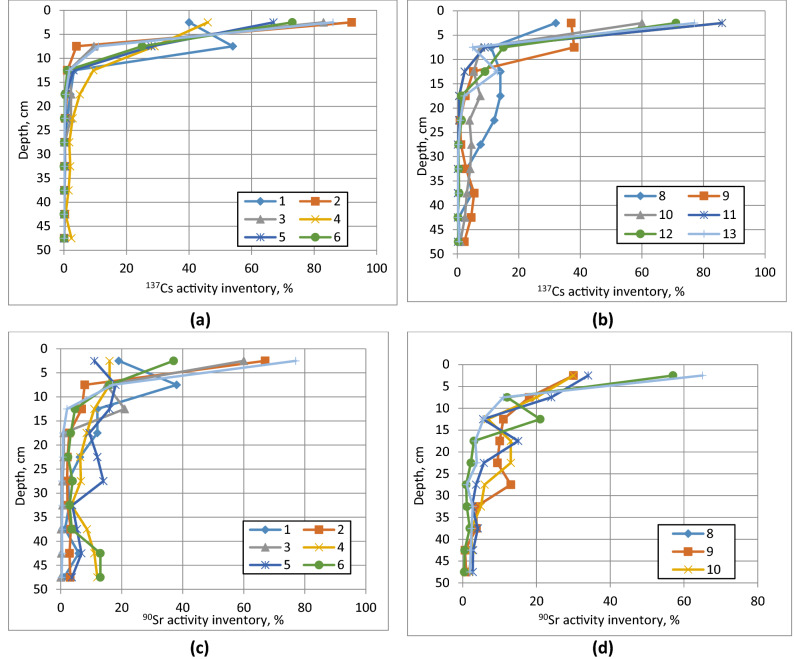
Vertical distribution of ^137^Cs ((**a**), (**b**)) and ^90^Sr ((**c**), (**d**)) in soils of landscape polygons of “Ecocenter” in the CEZ in 2020. The analytical error of ^137^Cs and ^90^Sr activity measurements is 20–30%. The landscape polygons (LP) no.1–4, 11 and 12 are situated within the glacial plateau; LP no.5, 7,8, 10 and 13 are situated within the terrace of the Pripyat River; LP no.6 and 9 are situated within the floodplain of Pripyat River. (Map showing location of sampling sites is given in Supplemental Information, Figure S.10.1).

Data on vertical distribution of ^137^Cs and ^90^Sr in soils of “landscape polygons” (experimental sites) in the CEZ in 2020 (Fig. 2) generally conform to the discussed above migration patterns, and show that for ^137^Cs more than 90% of activity inventory was concentrated in the upper 0–20 cm, while for ^90^Sr the same inventory was distributed in the upper 0–40 cm. In some instances, in addition to the maximum in upper 10–20 cm, a second peak of ^90^Sr was observed at 40–50 cm depth. In two locations (polygons 4 and 6) the second ^90^Sr peak migrated beyond the sampled 50 cm depth. Data of soil sampling carried out at landscape polygons in 2012 can be found in Kireev et al.^39^.

About 50% of the territory of the CEZ has depth to groundwater table (GWT) of 1.0–3.0 m and at about 20% of territory has depth to GWT of 3.0 m and more^21^. Since vertical distribution of ^137^Cs and ^90^Sr in soil has been slow (Fig. 2), there was potential for only relatively small amounts of fallout radionuclides (mainly ^90^Sr) from the topsoil to reach the groundwater system in locations with relatively deep (> 1.0 m) groundwater table during the time that has passed since the accident. The areas with a shallow groundwater table (< 1.0 m) in the CEZ are usually related to river floodplains with organic-rich meadows and wetlands. Such areas often represent groundwater discharge zones, which are characterized by an upward groundwater flow regime. This type of hydraulic regime may preclude entering of radioactive contaminants to the deep subsurface aquifers. On the other hand, the radioactive contaminants can be mobilized from wetland soils by upward groundwater flux and / or diffusive transport to wetland water, and enter nearby river networks by the surface runoff mechanism^22^.

### Contamination of the unconfined aquifer

**Figure 3:**
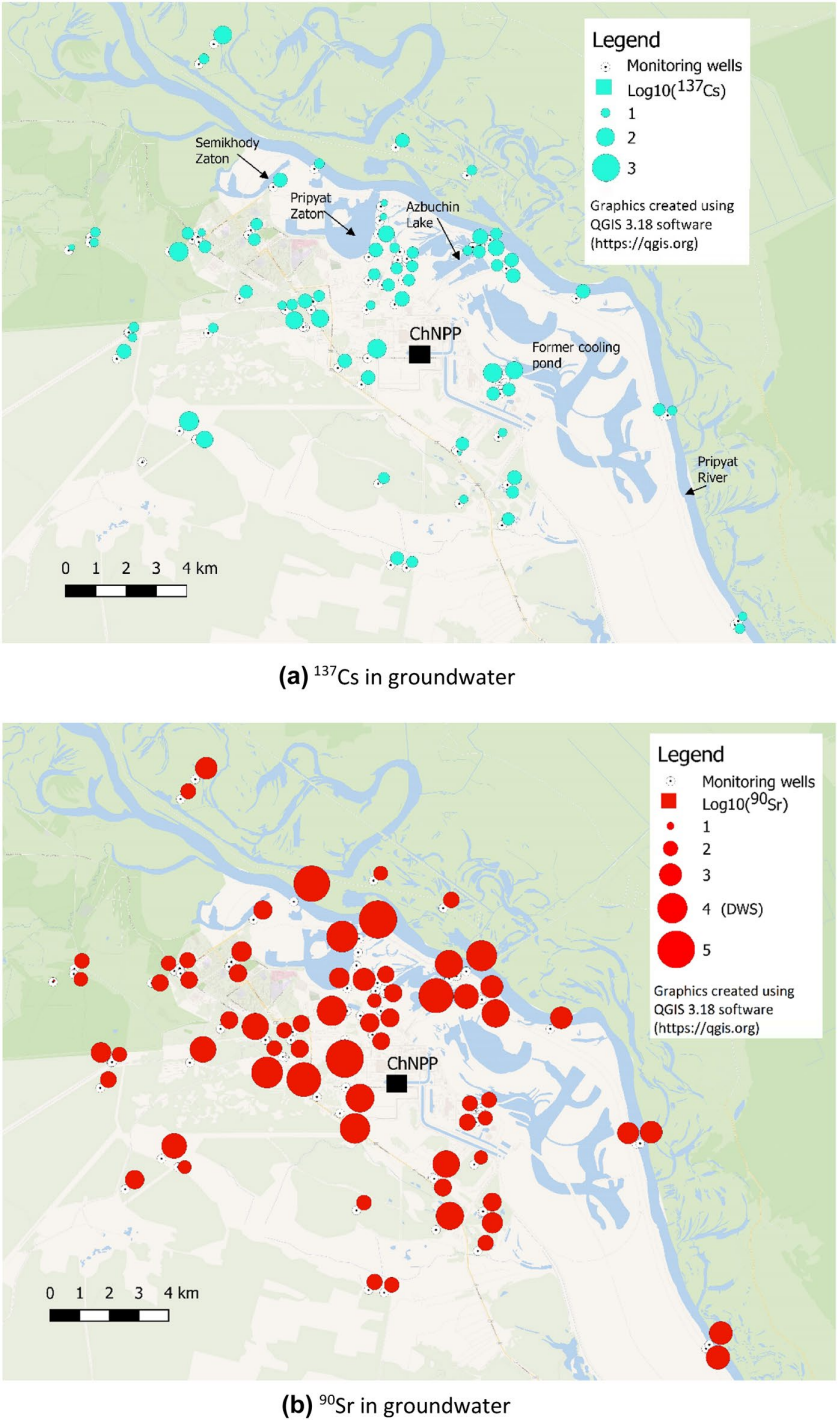
Distribution of ^137^Cs and ^90^Sr in groundwater of the 10-km zone of ChNPP in 2019 (Log_10_—transformed yearly-averaged concentrations in Bq m^−3^). The WHO drinking water standard for these radionuclides is 1 × 10^4^ Bq m^−3^.

The ^137^Cs concentrations in monitoring wells sampling the top unconfined aquifer in 2019 showed relatively low variability across the inner 10-km zone varying in the range 10–100 Bq m^-3^, which is below the WHO drinking water standard (DWS) of 10,000 Bq m^−3^ (Fig. 3a). No specific pattern is observed for ^137^Cs in groundwater in relation to the sources of contamination discussed above.

The ^90^Sr concentrations in groundwater in 2019 showed much higher variability, ranging from 10^2^ to 10^5^ Bq m^-3^, and exceeded the WHO DWS of 10 000 Bq m^−3^ in 16 of 87 wells. The highest levels of ^90^Sr are observed at waste dump sites and in monitoring wells influenced by seepage from surface water bodies containing contaminated bottom sediments (residual lakes in the former cooling pond, Pripyat Zaton, Semikhody Zaton and Azbuchin Lakes; see Fig. 3b). The ^90^Sr in groundwater does not correlate with ^137^Cs, which illustrates different migration mechanisms and mobility of these radionuclides in the subsurface^42^^,^^16^.

**Figure 4:**
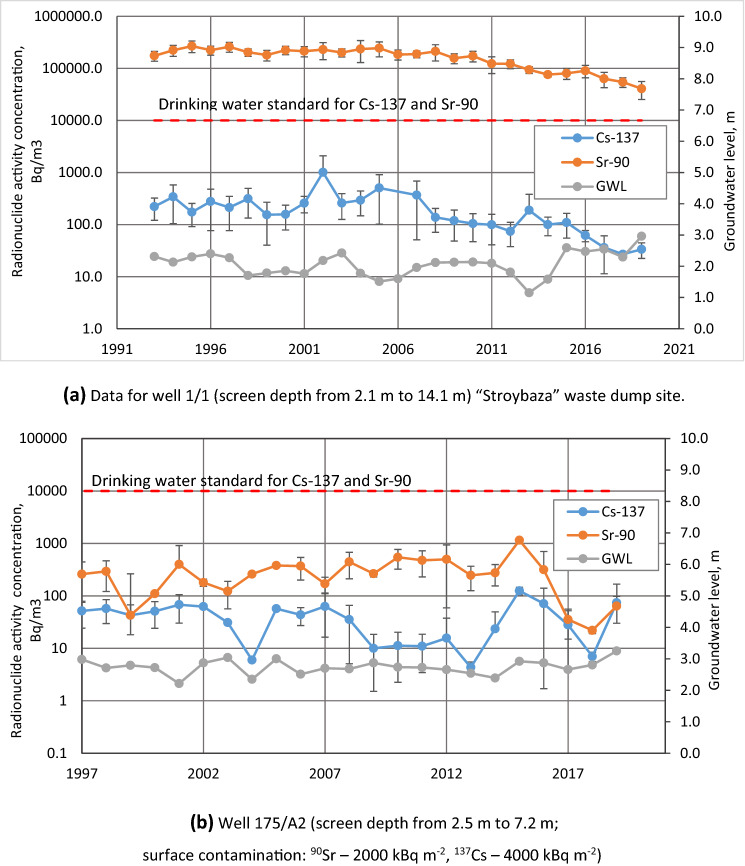
Groundwater monitoring data for (**a**) well 1/1 in the “Stroybaza” waste dump site and (**b**) in an area of high surface soil contamination in the 10-km zone of the ChNPP. Vertical bars represent standard deviation of data during a year.

**Figure 5:**
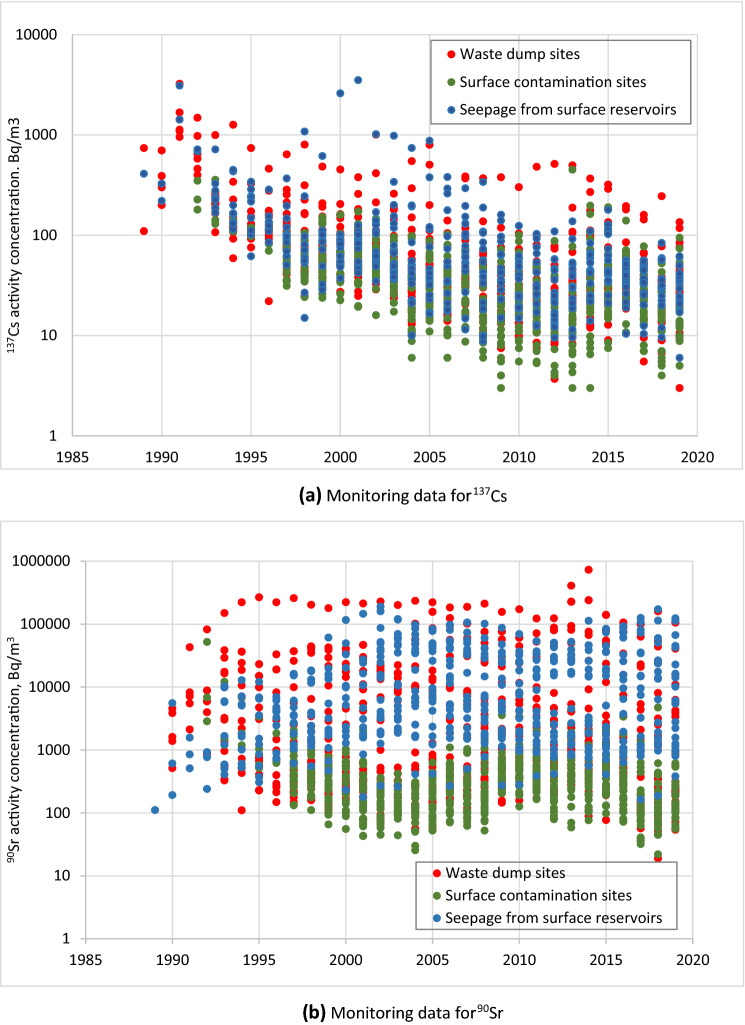
Time series of radionuclide activity concentrations in the unconfined aquifer for sites with different types of sources of groundwater contamination for the integral set of monitoring wells (monitoring data set is provided in Annex 6).

Example time series of ^90^Sr and ^137^Cs in monitoring well 1/1 located in the vicinity of the “Stroybaza” waste dump is shown in Fig. 4a (a map showing location of this and other wells mentioned below is provided in Supplemental information, Figure S10.3). Maximum levels of ^90^Sr of 2.3 10^5^ Bq m^−3^ in this well significantly exceed pre-accident background levels (i.e., 1–10 Bq m^−3^ based on data of^68^ and are indicative of ^90^Sr migration from buried wastes. The ^90^Sr data in this and most other wells of monitoring network in CEZ show increase (presumably caused by radionuclide release from fuel particles and breakthrough to groundwater system) during the first decade of observations after the Chernobyl accident followed by stabilization and tendency to decrease of activity concentrations in the majority of locations during the last decade indicating a gradually exhausted source term (Fig. 5b). A gradual decrease in 1998–2008 of mean ^90^Sr plume concentrations was observed also at an experimental site near trench no.22 at “Red Forest” waste dump site equipped with detailed multilevel monitoring well network^69^. Attenuation of ^90^Sr migration was explained by evolution of geochemical composition of groundwater (decrease of concentrations of Ca and other leached ions accompanied by an increase of pH) caused by humification of the organic matter (buried vegetation remnants) inside the waste trench, and of nutrient element uptake by roots of the pine forest growing on top of the trench^15^. Additional data on groundwater contamination at waste dump sites, showing similar trends, are presented in Supplemental information, Sect. 3.

An example time series of ^90^Sr and ^137^Cs in monitoring well 175/A2 sampling the upper part of the unconfined aquifer in the area with contaminated topsoil is shown in Fig. 4b. The ^90^Sr concentrations were relatively stable during the observation period and were 1–2 orders of magnitude less than the WHO DWS. The ^137^Cs concentrations showed a tendency to decrease during the first decade of observations, and their current range of about ~ 10–100 Bq m^−3^ is close to the pre-accident background levels from global nuclear weapons testing fallout. Similar trends in ^137^Cs concentration are observed for other monitoring locations in CEZ (see Fig. 5a). Conclusions on stabilization of ^137^Cs and ^90^Sr contamination levels in groundwater of  the CEZ during the last decade of observations are supported by Mann–Kendall statistical analysis of trends^4^ (see Supplemental Information Sect. 2.1 and Annex 5).

No immediate correlation is observed between radionuclide concentrations in groundwater and topsoil contamination level by Chernobyl fallout (see Supplemental Information, Figure S2.1).

The ^90^Sr concentrations in groundwater at waste dump sites often show clearly defined stratification with an upper part of the unconfined aquifer being much more contaminated than the lower part (see Supplement Information, Sect. 2.3, Figure S2.2 for more detail). Sampling at waste dump sites using auger drilling from the very top of the unconfined aquifer showed ^90^Sr concentrations up to 10^6^—10^7^ Bq m^-3^^17^ (lower levels of ^90^Sr in monitoring wells are explained by the depth-averaging effect of 12 m long screens of these wells). The ^137^Cs, on the contrary, usually shows decreasing time trends in groundwater and no or little variability with depth below the groundwater table, being close to the analytical detection limit for this radionuclide.

The typical range of actinide elements (Pu isotopes, ^241^Am) concentrations in groundwater of the CEZ in 2018–2019 was of order 0.1 to 1 Bq m^−3^ or less (see Supplemental information, Table S4.1). These concentrations were relatively stable over the observation time period being much lower than the WHO DWS of 1000 Bq m^-3^ for the sum of transuranium α-emitting radionuclides.

Low levels of radionuclides in groundwater in areas with topsoil contamination (see Fig. 5) are consistent with the data discussed above on low velocities of transport of fallout radionuclides in the soil profile.

### Seepage from the cooling pond and other contaminated water bodies

The cooling pond (CP) was the first site in the CEZ where high levels of groundwater contamination by ^90^Sr were detected in 1988–89. High mobility of ^90^Sr in groundwater seeping from the CP was caused by high seepage rates (due to a large difference in water level in the CP and in the adjacent Pripyat River) and by the low retention capacity of the sandy soil of the dam of the CP. Seepage of contaminated water from the CP used to be the major source of contamination of the Pripyat River by ^90^Sr until decommissioning of the CP in 2014^11,11^. The decommissioning strategy for the CP consisted in a partial drainage of the pond by lowering its water level (see Supplemental information, Figure S1.2). The decommissioning resulted in a significant decrease of seepage losses and ^90^Sr transport due to a drop of hydraulic head gradient between the CP and Pripyat River^30^.

Relatively high levels of ^90^Sr were observed in 2019 in monitoring wells influenced by seepage from residual lakes situated in the northern part of the former CP, as well as other water bodies containing highly contaminated bottom sediments, in particular in the vicinity of Pripyat Zaton, Semikhody Zaton and Azbuchin Lake (see Figs. 3b, 5b, and Supplement information, Sect. 5). Water levels in these water bodies are 2–3 m higher than the average annual water level in Pripyat River, which resulted in water seepage through the bottom and banks of these reservoirs towards the river. In the course of seepage, the surface water passed through contaminated bottom sediments containing FP. This could have caused additional leaching of radionuclides from FP to pore water, and resulted in groundwater concentrations of ^90^Sr, which were higher compared to historic contaminant levels in surface water^30^.

### Groundwater contamination at the Chernobyl Unit 4 site

A specific groundwater contamination ‘hot spot’ is the ChNPP Unit 4 ‘Sarcophagus’ (or ‘Shelter’). The potential sources of groundwater contamination here include the accident-related buried soil layer containing nuclear fuel materials (see Table 1) as well as possible leakage of the highly contaminated “block waters” located in the basement premises of the destroyed Unit 4^55,63^. The following groundwater concentrations were detected at the site in 2015^48,49^: ^137^Cs: from 300 to 7.2 10^4^ Bq m^−3^,^90^Sr: from 1000 to 1.8 10^6^ Bq m^−3^,^3^H: from 1000 to 8 10^5^ Bq m^−3^,transuranium element activity (Pu, Am, Cm isotopes): 300 – 700 Bq m^−3^. The monitoring data during last two decades show generally decreasing trends in ^137^Cs, ^3^H and transuranic elements, and fluctuating levels of ^90^Sr^48–50,55–57^. The intermittent increases and decreases of ^90^Sr are attributed to changes in groundwater pH and alkalinity (caused by groundwater interactions with concrete)^46,57^. The specific features of groundwater contamination at the ‘Sarcophagus’ are relatively high ^137^Cs concentrations. The possible explanation is that radioactive source materials here were enriched in ^137^Cs. In particular, the ^137^Cs to ^90^Sr activity ratios in “block waters” were in 2017 on average 10.2 while the maximum value was 326 (Odintsov and Han)^53^. This is much higher than the typical ^137^Cs:^90^Sr ratio of between 1 and 2 in soil of the 10 km zone^35^.

### Monitoring data for confined aquifers

Data for 1999–2019 for the Eocene aquifer, which is used for water supply of ChNPP, showed very low levels of ^137^Cs and ^90^Sr in water mostly in the range of 1 – 10 Bq m^−3^ (close to the detection limit for these radionuclides; see Supplemental information, Sect. 6). Similar data were reported also in a number of other much earlier surveys of this aquifer^21^.

The recent data presented here (Supplemental information, Sect. 6) support previous groundwater modeling analyses^10,66^, which suggested a large residence time of ^90^Sr from the sources of contamination to water wells exploiting the Eocene aquifer, allowing for ^90^Sr decay to safe levels. Similarly, there is no evidence of radioactive contamination of the lower confined aquifer in chalk deposits which provides the drinking water supply for Chernobyl Town. A discussion of residence times of groundwater and radionuclides in the confined and unconfined aquifers can be found in Supplemental Information, Sect. 7.

### Self-settler wells

**Figure 6:**
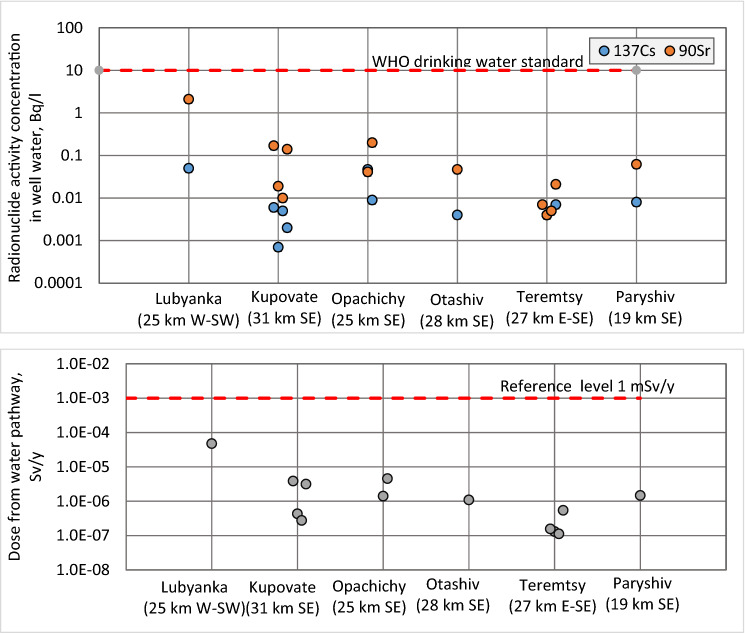
Radionuclide concentrations in the wells used by ‘samosely’ (self-settlers) in CEZ in 2019 and respective doses caused by drinking water consumption. (Map showing location of villages is given in Supplemental Information, Figure S10.2). Analytical errors are within the circular markers.

Data on contamination of water in dug wells to the unconfined aquifer used by ‘samosely’ (unauthorized self-settlers living in the CEZ, but outside the 10-km zone) is displayed in Fig. 6. The calculated doses via the drinking water pathway (assuming water consumption rate of 800 L y^−1^) are more than ten times below the (‘safe’) lower reference level of 1 mSv y^−1^ for ‘existing exposure situations’^29^.

### Off-site radionuclide transport to the Pripyat-Dnieper River system

The Pripyat River during the post-accident period showed decreasing trends of ^137^Cs and ^90^Sr in river water (Supplemental Information, Figure S9.1). This time dynamics was determined by radionuclide leaching rates from fuel particles, deepening of mobile forms of radionuclides in the soil profile and gradual depletion of activity sources in flooded soils and near-channel wetlands, which mainly contributed to contamination of the river water^22,42,45,70^. Declining trends in radioactivity of water are also observed following the Chernobyl accident for small rivers in the CEZ such as the Sakhan River and Uzh River^31,39,44^.

Declining exponential trends of radionuclide concentrations in rivers (without evident delayed peaks caused by groundwater transport from topsoil) were observed also following global nuclear tests fallout^67^.

**Figure 7:**
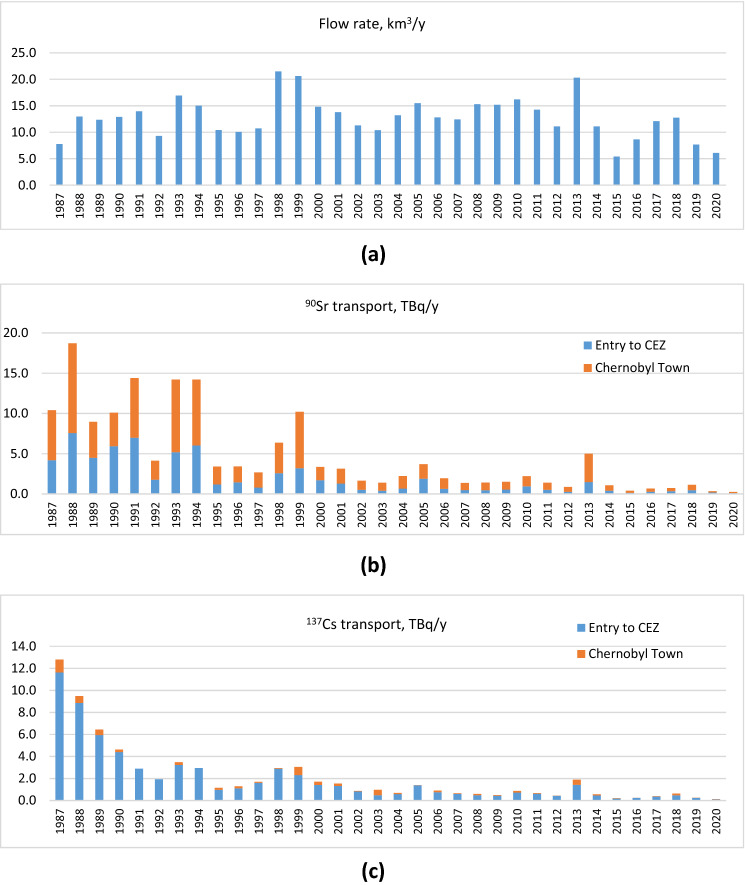
(**a**) Yearly water flow in the Pripyat River; (**b**), ^90^Sr, and (**c**) ^137^Cs budgets in the Pripyat River at the entry to CEZ. The additional amount of ^90^Sr and ^137^Cs added between entry to the Ukrainian CEZ and the observation point in Chernobyl Town outside the inner 10 km Zone is shown in orange bars. The additional ^137^Cs runoff from the 10 km Zone is a small proportion of the total entering Ukraine from Belarus, but this proportion is much more significant for ^90^Sr.

Figure 7 shows the total radionuclide fluxes transported by Pripyat River entering the Ukrainian CEZ at the Ukraine-Belarus border and the amounts added by sources in the 10 km zone from monitoring at Chernobyl Town at the downstream edge of the 10 km zone. Contamination of the Pripyat River water by ^137^Cs during the post-accident period was mainly (80—95%) formed in areas upstream of CEZ, not by leaching from contaminated soils and wastes at the site or in the inner 10 km zone (Fig. 7). On the contrary, the main sources of ^90^Sr to the Pripyat River (50 to 70%) are located inside the CEZ. The balances of ^90^Sr in the Pripyat River indicate that highest radionuclide inputs to surface water are associated with high flow events (e.g., 1998–99 and 2013; see Fig. 7) due to flooding by river of floodplain soils and direct leaching to surface water.

Maximum yearly averaged activity concentrations in the Pripyat at Chernobyl Town during the last decade were 180 Bq m^-3^ of ^90^Sr and 76 Bq m^-3^ of ^137^Cs, significantly below the WHO drinking water standard of 10,000 Bq m^-3^ of these isotopes.

### Natural barriers control long term groundwater impacts

The observed radionuclide mobility in soils and groundwater of the CEZ (decreasing in the order ^90^Sr > ^137^Cs > Pu > ^241^Am) correlates with the sorption distribution coefficient (K_d_) values for these radionuclides,^16,42,44^, which evidences the principal importance of sorption in controlling the migration process.

Stable or declining trends of ^90^Sr in groundwater are observed in most monitoring locations except for a few wells where the leading edge of sub-surface plumes originating from water bodies with contaminated bottom sediment passes monitoring stations (see Supplemental document, Figure S5.1).

The decreasing trends of ^90^Sr concentrations in groundwater can be explained by the combined effect of natural attenuation processes which include exhaustion of the higher-intensive source of ^90^Sr fallout in soils in the form of relatively fast dissolving oxidized fuel particles^37^.

Additional factors include stabilization of the geochemical regime of groundwater below the waste dumps due to maturation of organic matter buried inside the waste dumps^15^. Involvement of ^90^Sr and ^137^Cs (which are chemical analogs of major plant nutrient ions—Ca and K, respectively) in bio-cycling in the ‘soil plant’ systems played a key role in retention of radionuclides in the topsoil rooting layer and in waste burials overgrown by trees^51,51^. The highest vertical ^90^Sr migration rates in soil during the post-accident period have been observed in “dis-equilibrated ecosystems”, for example in areas of wildfires^28^, where radionuclide cycling in the soil–plant system has been disrupted. Other relevant attenuation mechanisms include radioactive decay and dispersion during subsurface transport.

Management interventions have led to better protection of groundwater from radioactivity sources, including the decommissioning of the cooling pond, which has resulted in decrease of seepage of contaminated water to Pripyat River from this reservoir, and an increase of the thickness of the unsaturated zone at the ChNPP industrial site including the ‘Sarcophagus’ (see Supplemental information, Figure S1.2c). Completion of the NSC arc on the ‘Sarcophagus’ in 2016 has led to better protection of facility and its vicinity from infiltration of atmospheric water. This resulted in a decrease of volume of extremely radioactive “block waters”, which have posed a risk of leakage to the surrounding geological environment^53^.

A clear understanding of the natural or artificial process causing small concentrations of generally low-mobility radionuclides such as ^137^Cs, Pu and Am isotopes, in groundwater of the unconfined aquifer in the CEZ remains an open question^16^. Potential explanations include colloid transport^1,38,60^, depression focused recharge^5,65^, or alternatively, the facilitated transport could have been caused by imperfect well design (e.g., cross-contamination from topsoil during drilling, vertical flow along the poorly sealed well casing)^20^. The monitoring data presented here imply, however, that facilitated migration mechanisms discussed above, if present, are responsible for relatively low levels of groundwater contamination by ^137^Cs and actinide elements. Small amounts of ^137^Cs in groundwater which decreased with time were also observed following the Fukushima accident^62^.

**Figure 8:**
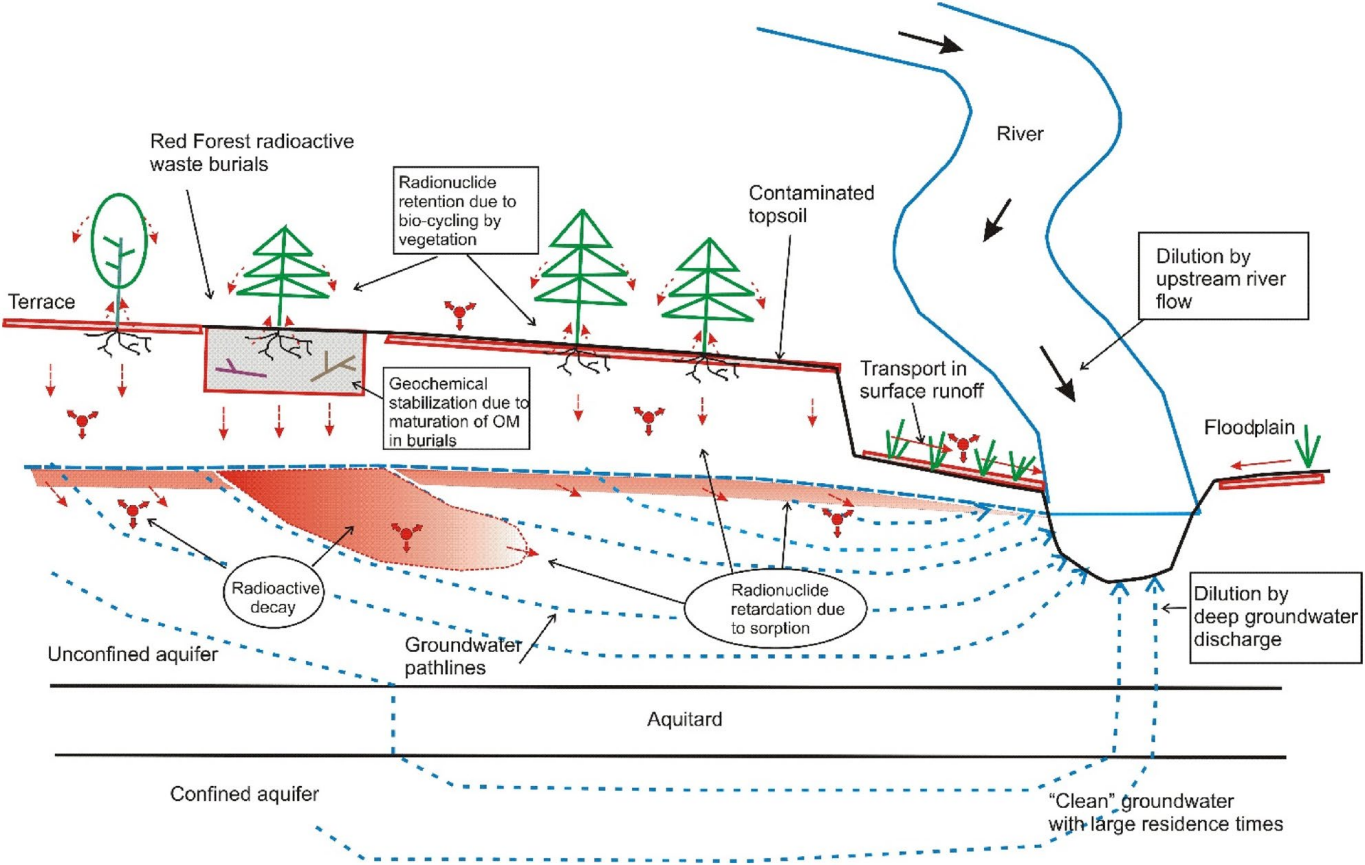
Conceptual scheme of natural attenuation processes in groundwater and surface water of the CEZ.

The results of this long-term study, representing the most comprehensive evaluation of radioactivity in groundwater to date, confirm early modelling predictions that the groundwater transport of radionuclides from waste dumps and contaminated watershed topsoil^12,14,52^ and from the ‘Sarcophagus’ ^40,63^, results in generally negligible risks to populations downstream of the CEZ, while the deep aquifers used for water supply are well protected by geological barriers^10,66^. The key factor is radioactive decay during retardation by sorption groundwater transport in subsurface (vadose zone, aquifers) from sources of contamination to receptor points. Radionuclide concentrations in groundwater are further attenuated due to mixing process at discharge contours (water supply wells, rivers) where a relatively small fraction of discharge is typically composed from “younger” waters that can potentially carry radioactivity (see Supplementary Information, Sect. 7). An important additional dilution effect in surface water comes from mixing of groundwater discharge with the clean upstream river water, which originated in the non-contaminated part of the river basin (Fig. 8).

Thus, monitoring data collected in CEZ during the last 35 years, presented in full for the first time here, shows that the natural attenuation process complemented by the geological settings and remedial and decommissioning measures result in generally favorable trends in contamination of groundwater and surface water, which are consistent with *a-priori* modeling predictions. Off-site risks via the Pripyat River, to self-settlers and to the confined aquifers which provide water to the NPP and to Chernobyl Town are low. This analysis of 35 years’ data supports the Monitored Natural Attenuation strategy for groundwater in the CEZ. The long-term sources of groundwater contamination at the” Sarcophagus” site, at radioactive waste dumps, and in the vicinity of lakes in the 10-km zone of ChNPP containing highly contaminated bottom sediments still require ongoing monitoring and assessment.

## Methods

### Groundwater monitoring program of “Ecocenter”

The groundwater monitoring program in the 30-km zone is carried out by the State Specialized Enterprise (SSE) “Ecocenter”. The groundwater monitoring constitutes a part of a comprehensive program of radiation monitoring of the environment in the Ukrainian part of the 30-km zone, which includes air dose rate monitoring, radioactive aerosol concentrations, soils, surface water bodies and biological species. Radiation monitoring in the 30-km zone was performed from the early days following the accident. These works were carried out first by the Production Association “Kombinat” (which was responsible for Chernobyl accident mitigation measures in the 30-km zone), followed by the “Pripyat” Scientific and Production Association, then by the State Enterprise for the Regional Environmental Monitoring “RADEK”, and since 2000 until the present time, by the SSE “Ecocenter”. The integrated database of radioactivity measurements for the whole post-accident period is maintained by the SSE “Ecocenter”. This database is the main source of monitoring data which are presented and analyzed in this article.

### Groundwater monitoring network

The groundwater observation well network in the 30-km zone is primarily tailored to the unconfined aquifer in Quaternary deposits, which has suffered most from radioactive contamination.

Development of the groundwater monitoring system proceeded through several stages. The first targeted regional-level groundwater monitoring well network at ChNPP site was drilled in 1990. At that time, observations were carried out using 60 wells. The monitoring wells often had large 12-m screens (wells of the K-series) leading to vertically-averaged samples. In 1995–1996 the groundwater monitoring system of 30-km zone was complemented by a number of multi-level observation well clusters with 1–2 m long screens providing samples from different aquifer depth intervals.

At the present time, the monitoring well network of the “Ecocenter” for the unconfined aquifer in Quaternary deposits consists of 145 monitoring wells (see Supplemental information, Figure S10.3). This number includes object-level monitoring networks of engineered radioactive waste disposal sites “Buryakovka”, “Podlesny” and “3^rd^ Stage of ChNPP”.

In addition, the quality of water is monitored in the deep aquifers: confined aquifer in Eocene deposits exploited by Pripyat Town water wells, and in the confined aquifer in Cretaceous fractured chalk extracted by Chernobyl Town water supply wells.

Groundwater sampling is carried out also from the dug wells used by unauthorized self-settlers in Chernobyl exclusion zone (“samosely”) for domestic use at their residential locations (Chernobyl, villages Kupovate, Paryshiv, Teremtsy).

The monitored parameters include: groundwater level; volumetric activities in water of ^137^Cs, ^90^Sr and transuranic elements (^238^Pu, ^239 + 240^Pu, ^241^Am).

Sampling frequency from different locations varies from 1 time per quarter to 1 time per year. The total number of groundwater samples collected per year is currently 720. All collected samples are analyzed for ^90^Sr, and about 60% of samples are analyzed for ^137^Cs. About 20 samples per year are analysed for transuranic elements.

## Field sampling and analytical methods (“Ecocenter” laboratory)

### Groundwater sampling and analysis

The groundwater monitoring wells are purged before sampling until pumped water is visually free from suspended particles. Upon collection of samples, flasks with water are filled to the brim and tightly sealed to avoid contact of water with atmospheric air. Analytical studies are carried out on unfiltered non-acidified samples. The ^90^Sr in groundwater samples (typically 1 l volume) is determined by a radiochemical method^24^. The ^90^Sr is separated from the solution by precipitation of carbonates (by the addition of sodium carbonate and sodium hydroxide). The chemical yield of ^90^Sr is determined by the weight method (by adding a stable Sr label). The activity of ^90^Sr in the precipitate is determined using beta-radiometer NRR-610 (TESLA). The analytical error of ^90^Sr determination is (depending on activity of water, for 1 l sample): 0.01—0.1 Bq l^−1^–20–30%,0.1–0.5 Bq l^−1^–10–20%,˃ 1 Bq l^−1^–˂10%. Here and below values of the total analytical errors are provided (corresponding to the 95% confidence level) taking into account the errors of measuring instruments (balances, pipettes, spectrometers), errors allowed during sample preparation and its radiochemical processing etc. The counting time varied from 4 to 12 h (8 h on average). The value of minimum detectable activity (MDA) is 0.1 Bq/sample with a counting time of 15 h.

The ^137^Cs is extracted from solution by filtration of a water sample (typically 20 l volume) through an ion-exchange resin (“ANFEG”, manufactured by “Ecosorb” LLC, Russia) followed by determination on an ion-exchange material using SEG-002 “AKP-P” gamma-spectrometer equipped with a high purity germanium detector (“Atomprylad”, Kyiv). The chemical yield of ^137^Cs was estimated by analyzing “model samples”. “Model samples” were prepared by adding a certain amount of a standard radioactive solution of ^137^Cs to water samples with a similar chemical composition to studied samples. The chemical yields thus obtained was close to 100%. The analytical error of ^137^Cs determination is (depending on activity of water, for 20 l sample): 0.005–0.01 Bq l^−1^–20–30%; 0.01–0.05 Bq l^−1^–10–20%; > 0.05 Bq l^−1^–˂10%. The MDA value was 0.1 Bq/sample with a counting time of 72 h.

Isolation of ^238,239 + 240^Pu isotopes from the water sample (typically 40 l volume) is carried out on anion exchange resin AV-17 (http://smoly.com.ua/silnoosnovnyiy-anionit-av-17-8) by selective sorption in the form of nitrate complexes^19^. Elution of plutonium from the resin is carried out with a weak solution of hydrochloric or nitric acid. Isolation of ^241^Am from the samples is also performed on anion exchange resin AB-17. Elution of americium from the resin is performed with a mixture of hydrochloric acid and methanol. The sample for alpha-spectrometric determination of ^241^Am and Pu isotopes is prepared from the obtained solutions by the method of electrolytic deposition on a stainless-steel plate. The measurements are carried out using multi-channel alpha spectrometer α12 based on silicon ion-implanted detectors SIID-12 manufacturer Baltic Scientific Instruments.

The chemical yield of plutonium isotopes (^238–240^Pu) was determined by adding the ^242^Pu label. Analyses of ^241^Am utilized the ^243^Am label. The value of chemical yield of actinide elements for samples with different chemical composition and level activity varied from 30 to 90%. Information on certified reference solutions of radioactive labels is provided in Table 2.

**Table 2:** Certified reference solutions of radioactive labels used in analytical studies of actinide elements.

Title (type) of reference solution	Radionuclide	Activity (as of production date), kBq	Manufacturer	Certificate
Plutonium-242 Alpha Standard Solution	^242^Pu-	2.3600E−01 (4.20E−02 kBq/g × 5,616 g)	Eckert&Ziegler Nuclitec GmbH (Germany)	CO-401853
Americium-243 Gamma Standard Solution	Am-243	3.9500E+00 7.90E−02 kBq/g × 50.025 g)		CO-405217

The analytical error of actinide element determination is (depending on activity of water, for 40 l sample): 0.0001–0.002 Bq l^−1^–20–30%; 0.002–0.008 Bq l^−1^–15–20%; > 0.008 Bq l^−1^– <15%.

The MDA for ^238-240^Pu and ^241^Am by alpha spectrometric method is 0.0005 Bq/sample with counting time of 3–7 days.

### Soil sampling and analysis

The soil sampling in order to study vertical migration of radionuclides in soils of ‘landscape polygons’ was carried out from 1.5 (lengths) × 0.8 (width) × 0.5 m (depth) dug pits in soils of experimental sites. At each site soil sampling was carried out using the “envelope method” in 5 locations of a 5 m × 5 m square (in square corners and in the middle). Once pits were prepared, the soil sample collection was performed from the vertical wall of pit in each location from its bottom in upward direction. The soil sampling depth interval was 5 cm. Soil samples collected from the same depths in 5 locations were mixed to produce one composite sample, which was directed to further analyses. Soil sampling was done using a cylindrical sampler with a diameter of 50 mm. An average weight of the dry composite soil sample was 1120 g.

The gamma-spectrometric analysis of soil samples in order to estimate ^137^Cs activity used the gamma-spectrometric complex Ortec-919 Spectrum Master with detectors Canberra-GC2518 and Ortec-GMX 40P4-83RB. The minimum detected activity (MDA) of these detectors is 0.5 and 1 Bq per sample respectively (for measuring time of 12 h or more).

The ^90^Sr activity in soil was measured directly on collected soil samples using the beta-spectrometry method^6,18^. Measurements were carried out using the SEB-01–150 scintillation beta-spectrometer with the AKWin software (Atom Komplex Prylad, Ukraine) (http://www.akp.com.ua/). The MDA for ^90^Sr for a standard geometry sample is 2.7 Bq per sample (for exposure time 2 h).

### Balance calculations of hydrologic transport of radionuclides by the Pripyat River

Calculations of yearly transport of radionuclides by the Pripyat River at the entry to CEZ (Ukrainian state border with Belarus) and at the observation point in Chernobyl Town (at the downstream edge of the 10 km zone) are based on data of regular observations of river levels, flow rates and radionuclide (^90^Sr, ^137^Cs) activity concentrations in river water.

Surface water sampling was carried out from boat or from ice using sampling device with the 1 l volume. Samples were taken from at least 10 vertical depth profiles evenly spaced across the width of the river. At each vertical profile two points were sampled: near the water surface (at the 20% depth of river in respective location) and near the bottom (at the 80% depth). The water flow velocity measurements were carried out simultaneously. The collected 1 l water samples were merged to produced composite sample with a volume of about 20 l, which was directed to the laboratory for analytical studies.

During low-flow seasons (summer, autumn) hydraulic parameter measurements and water sampling is carried out with frequency 2–4 times per month. During high flow events (spring floods, extreme rainfall events) sampling is carried out with 3–5-day frequency.

### Dose calculations for groundwater pathway for self-settlers (‘samosely’)

Dose calculations for self-settlers in the Chernobyl zone from drinking water pathway are based on assumptions that 100% of drinking water supply comes from domestic wells, and that the water ingestion rate is 800 L/year ^58^. The dose calculations employ the dose coefficient of 1.3E-8 Sv/Bq for ^137^Cs, and 2.8E-8 Sv/Bq for ^90^Sr^29^.

## Supplementary Information

Supplementary Information 1.

Supplementary Information 2.

## Data Availability

All main monitoring data and other relevant data are available in the supplementary materials. Any additional data and materials are available on request from the corresponding author.
