# Supplementary material for: Natural attenuation processes control groundwater contamination in the Chernobyl exclusion zone: evidence from 35 years of radiological monitoring
Source: Sci Rep. 2022 Oct 29;12:18215. doi: 10.1038/s41598-022-22842-5 (PMC9617897; doi:10.1038/s41598-022-22842-5)
Supplement: Supplementary file 2 — Supplementary Information 2. [file 41598_2022_22842_MOESM2_ESM.docx]

SUPPLEMENTAL INFORMATION

Information on site geology and hydrogeology, and on radioactive contamination of groundwater and surface water in Chernobyl exclusion zone

**Contents**

[Section 1. Chernobyl zone geology and hydrogeology 3](#_Toc106615165)

[Section 2. Analysis of time trends and additional monitoring data on radionuclide distribution in groundwater in the unconfined aquifer in Quaternary deposits 9](#_Toc106615166)

[Section 3. Detailed studies of groundwater contamination at radioactive waste dump sites 14](#_Toc106615167)

[Section 4. Data on groundwater contamination by Pu isotopes and ^241^Am 17](#_Toc106615168)

[Section 5. Groundwater contamination caused by seepage from surface water bodies 18](#_Toc106615169)

[Section 6. Groundwater monitoring data for deep confined aquifers in CEZ 20](#_Toc106615170)

[Section 7. Groundwater dating studies and estimation of residence times of groundwater and radioactive contaminant (^90^Sr) in subsurface 22](#_Toc106615171)

[Section 8. Groundwater monitoring data for water supply wells used by ‘samosely’ in the Chernobyl exclusion zone 25](#_Toc106615172)

[Section 9. Data on ^90^Sr and ^137^Cs concentrations in the Pripyat River water 26](#_Toc106615173)

[Section 10. Supplemental maps 27](#_Toc106615174)

[Supplemental References 31](#_Toc106615175)

**Annexes to supplemental information (in Excel format) provide rough monitoring data:**

SUPPLEMENTAL INFORMATION_Annex1 - Compilation of groundwater monitoring data collected by the SSE "Ecocenter" in Chernobyl exclusion zone in 2018-2019 (unconfined aquifer in Quaternary deposits)

SUPPLEMENTAL INFORMATION_Annex2 - Compilation of groundwater monitoring data collected by the SSE "Ecocenter" in Chernobyl exclusion zone in 2018-2019 (confined aquifers)

SUPPLEMENTAL INFORMATION_Annex3 - Compilation of surface water monitoring data for Pripyat River collected by the SSE "Ecocenter" in Chernobyl exclusion zone in 1987-2020

SUPPLEMENTAL INFORMATION_Annex4 -Data (table format) on ^137^Cs and ^90^Sr distribution in soils of ‘landscape polygons’ of Chernobyl exclusion zone

SUPPLEMENTAL INFORMATION_Annex5 - Results (table format) of Mann-Kendall statistical analysis of trends of ^137^Cs, ^90^Sr activity concentrations and groundwater levels in monitoring wells in CEZ

SUPPLEMENTAL INFORMATION_Annex6 – Groundwater monitoring data of SSE “Ecocenter” for the period 1989 - 2019 used as an input for Mann-Kendall statistical analysis of trends

# Section 1. Chernobyl zone geology and hydrogeology

The ChNPP is situated in the middle reaches of the Pripyat River, which is a tributary of the Dnieper River. The 30-km zone is situated within the territory of the physio-geographical provinces of the Kiev and Belorussian Polesye (Woodlands). It is characterized by a moderately-continental climate with warm summers and rather mild winters. The mean annual temperature is +7^0^C, precipitation ranges from 450 to 750 mm y^-1^ (average 580 mm y^-1^), while open water evaporation is about 400 mm y^-1^. The territory of the CEZ is characterized by relatively flat topography. The surficial geological formations are composed of fluvioglacial and alluvial Quaternary (Middle to Late Pleistocene) sandy deposits. Before the accident, about half of the territory (45%) was covered by pine forests, as well as by mixed deciduous forests. The rest of the territory was occupied by agricultural fields (30%) and meadows (10%) (Davydchuk, 1996; Bondarkov t al., 2011).

The territory of the 30-km zone is covered by a dense network of rivers, streams, and agricultural drainage channels which form tributaries to the main water courses, the Pripyat and Uzh Rivers. The density of the hydrographic network in the CEZ is from 0.3 - 0.4 km km^-2^. The runoff coefficient to surface water (i.e., ratio of annual watershed precipitation to river runoff) is 10-30% (Galuschenko, 1991).

Below we will briefly describe the geological structure and hydrogeological conditions of the study area. The groundwater monitoring studies in the CEZ were mainly focused on the 10-km zone of the ChNPP, which contains the highest levels of topsoil contamination by fallout fuel particles and main radioactive waste storage sites, so most attention below is given to this central area of the CEZ.

**Geomorphological settings**

The terraces of rivers that cross the CEZ (Pripyat, Uzh and their tributaries) form an alluvial plain, which encompasses 30-40% of the territory and includes the floodplains (absolute elevations 105-110 m a s.s.l), the first above-floodplain terrace (elevations 110-120 meters a.s.l.) and second above-floodplain terrace. The second terrace of the Pripyat River occurs in the form of residual outcrops within the first terrace. The water dividing area of the Pripyat and Uzh rivers is formed by the Chernobyl- Chistogalovka moraine-fluvioglacial plateau (absolute elevations 120-165 meters a.s.l). The territory of the ChEZ has numerous poorly drained areas with swamps and wetlands, which are situated both in the watershed areas and within the river terraces and floodplains. Flat ground surface and high permeability of surficial deposits creates conditions where infiltration significantly exceeds surface runoff (Davydchuk, 1996).

The soil cover is most often represented by sol-podzol soils having low content of clay particles, humus (0.5-2%), and low cation exchange capacity (typical range 3.5-10 meq (100g)^-1^). These soil properties and their slightly acidic reaction (pH of water extracts in the range 4 – 5.5) creates conditions for potentially high mobility of fallout radionuclides in the soil profile. Acidic peat bog soils are common within land areas with lower elevations. Grassland soils with a thin fertile layer are common in the floodplains (Davydchuk, 1996; Bondarkov et al., 2011).

**Geological structure**

The studied territory is located within the North-western slope of the Ukrainian Shield. The crystalline basement (Precambrian rocks) in the ChNPP plant site lies at a depth of about 200 m. The sedimentary cover consists of marine and continental rocks of all systems of Mesozoic and Cenozoic erathems, which slightly dip to the East and Southeast. The geological structure of the sedimentary cover (from bottom to top) are as follows. The Upper and Middle Jurassic formations (J_3_) are represented by sands, clays, marls, siltstone, and limestone. Chalk deposits atop the Jurassic formations are represented by three suites: Cenomanian suite formations (K_2_cm) consist of water-saturated fissured sandstone and sands overlain by weakly permeable marl-chalk formations of Turonian, Coniacian and Santonian suites (K_2_t-cn-st).The total thickness of the chalk formation is about 100 m. Chalk deposits are overlain by saturated glauconite-quartz sands of Kanev (P_2_kn) and Buchak (P_2_bc) Eocene suites above which lie low permeable carbonate clay-siltstones and siltstone-clays (frequently referred to as marls) of Kiev suite of Eocene (P_2_kv). Sandy alluvial Neogene (N_2_) and Quaternary formations of Pleistocene and Holocene (Q_1-4_) lie atop the Kiev formations. The thickness of Quaternary alluvial formations is about 25-30 m (Dzhepo and Skalskyy, 2002).

Of most interest in the context of this article are the Quaternary formations in Pliocene and Pleistocene – Holocene sediments (Figure S1.1). These sandy deposits host the unconfined aquifer where migration of radionuclides of accidental release occurs from radioactivity sources (contaminated topsoil, waste dumps) to the surrounding geological environment.

The upper part of the Quaternary formation is composed of Upper Pleistocene – Holocene sandy deposits. Below is located the non-stratified continental horizon of the Pliocene – Middle Pleistocene age (see Figure S1.1). These sediments are sub-divided into aeolian and alluvial suites. The thickness of the Upper Pleistocene alluvial suite represented by inter-bedded layers of sands and silts is 9-12 m. The thickness of aeolian deposits (resting upon the alluvial suite) varies from several centimeters up to 5-7 m (in dunes) (Matoshko et al., 2004).


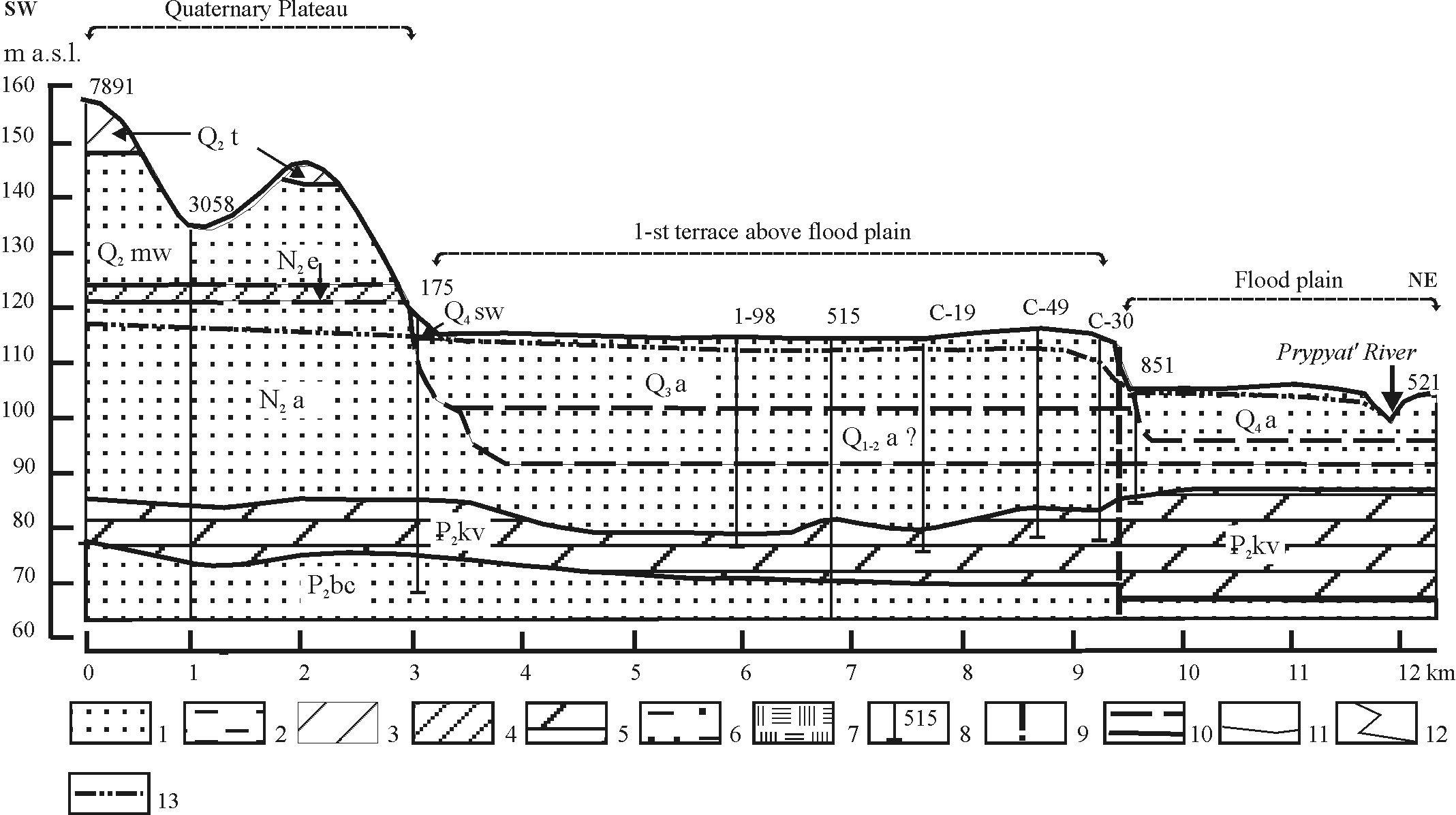


Figure S1.1. Geologic SW – NE cross-section of the ChNPP site from (Matoshko et al., 2004)
Legend: 1 – sands, 2- silts, 3 – basal till, 4 – clay, 5 – marl, 6 – inter-bedding of sands and silts, 7 – peat and peaty sand, 8 – boreholes (numbered), 9 – inferred fault, 10 – boundaries between suites: supposed (upper) and established (lower), 11 - boundaries between depositional facies, 12 – facial replacement, 13 – groundwater level (generalized). Indices: Q4 - Holocene, Q3-4 - Upper Pleistocene - Holocene unstratified, Q3 - Upper Pleistocene, Q1-2 - Lower Pleistocene - Middle Pleistocene unstratified, N2 - Pliocene, P2 – Eocene; kv - Kyiv, bc – Buchack. Genetic types of deposits: a - alluvial, mw – melt-water, eol - aeolian, e - presumably waste mantle, sw – slopewash. Facies: ob – overbank, ch – channel, a-ch – abandoned channel.

Quaternary sandy deposits usually have bulk density of 1.6 - 1.8 g cm^-3^, and porosity of 33 -42%. The cation-exchange capacity (CEC) of eolian deposits is usually less than 1 meq (100 g)^-1^, while the alluvial and fluvio-glacial deposits have CEC values of 2 - 10 meq (100 g)^-1^ (Matoshko et al., 2004; Bugai et al., 2012a).

Alluvial deposits are mostly composed of quartz with admixtures of feldspar (up to ~10 %). Their clay fraction is composed of kaolinite, montmorillonite and hydromica (illite), as well as finely-dispersed calcite, quartz, and amorphous ferrous oxides. In eolian sands the clay-size fraction is almost totally composed of finely-dispersed quartz (Kuznetvov, 1973; Olkhovik et al., 1992; Matoshko et al., 2004).

**Hydrogeological conditions**

The following main aquifers are present within the 30-km zone (from top to bottom): (1) the unconfined aquifer in the Quaternary sandy deposits, (2) the confined aquifer in the Eocene sandy deposits, and (3) the confined aquifer in the Cretaceous chalk deposits (Dzhepo and Skalskyy, 2002). The first two aquifers represent the zone of a most intensive “atmospheric water – groundwater – surface water” exchange, and these aquifers are discussed in more detail below.

Unconfined aquifer in Quaternary deposits

The unconfined aquifer is hosted by Pliocene and Pleistocene – Holocene sandy deposits. The thickness of the saturated stratum at ChNPP site usually is 20 - 30 m. Hydraulic transmissivity of the unconfined aquifer is estimated in the range 40-400 m^2^day^-1^ (in watershed areas, the upper aquifer strata may be combined with Neogene formations forming up to 90 m total thickness).

The regional flow in the unconfined aquifer at ChNPP site is directed to the Pripyat River and its tributaries. Hydraulic head gradients constitute 0.001 - 0.003 (Figure S1.2-a,-b). The real groundwater flow velocity in the in the unconfined aquifer (eolian sand layer) was estimated from natural gradient tracer tests conducted at experimental polygon Pilot Site in “Red Forest” waste dump site at ~10 m y^-1^ (this experimental site is situated within the first terrace of Pripyat River 2.5 km SW from ChNPP) (Bugai et al., 2012a).

The depth to the groundwater table in the floodplain river areas is usually 0 – 2m, in river terrace areas up to 5 – 7 m, and in water divide areas (glacial plateau) up to 14 - 17 m.

The main inflow of meteoric infiltration water to the unconfined aquifer usually occurs in spring following the snowmelt. Intensive rains in spring, summer or autumn can also provide significant inflow of infiltration water to the aquifer (Bugai et al.,2012a). The amplitude of seasonal fluctuations of ground water level is 0.5-1 m within the watershed areas, 1- 1.5 m within the river terraces, and 1-2 m or more within the river floodplain areas.

Infiltration recharge rate to the unconfined aquifer was estimated by means of calibration of the regional groundwater flow model of the CEZ (Skalskyy and Kubko, 2000): - 150-250 mm y^-1^ at river floodplain areas; - 50-150 mm y^-1^ at first river terrace with undisturbed (natural) hydrogeology conditions; - 200-300 mm y^-1^ at first river terrace within the area with waste dump sites (removed topsoil layer, spare vegetation cover); -30 – 70 mm y^-1^ in the water divide forested areas of the Uzh and Pripyat River (fluvio-glacial plateau). The above infiltration recharge rate value for the waste dump area is in good agreement with the infiltration estimate of 200 ± 50 mm y^-1^, or 40 ± 5% of the total amount of atmospheric precipitation obtained by the water table fluctuation method (mean value for 2001-2003) at an experimental site in the “Red forest” (Bugai et al., 2012a).

Groundwater in the unconfined aquifer at the ChNPP site usually is predominantly of the hydrocarbonate (sometimes hydrocarbonate-sulfate) and calcium-magnesium type with total dissolved solids content varying from 0.1- 1 g l^-1^ and pH≈6.0-6.5 (Dzhepo and Skalskyy, 2002; Sobotovich and Olshtynsky, 1991). Groundwater geochemistry at waste dump sites can be influenced by organic substances decomposition processes (e.g., litter, vegetation remnants) inside the waste burials and by leaching of chemical constituents to groundwater. This leads to increase of ion (calcium, potassium, hydrocarbonate) concentrations in groundwater downstream of the trenches (Bugai et al., 2012a,b).

| 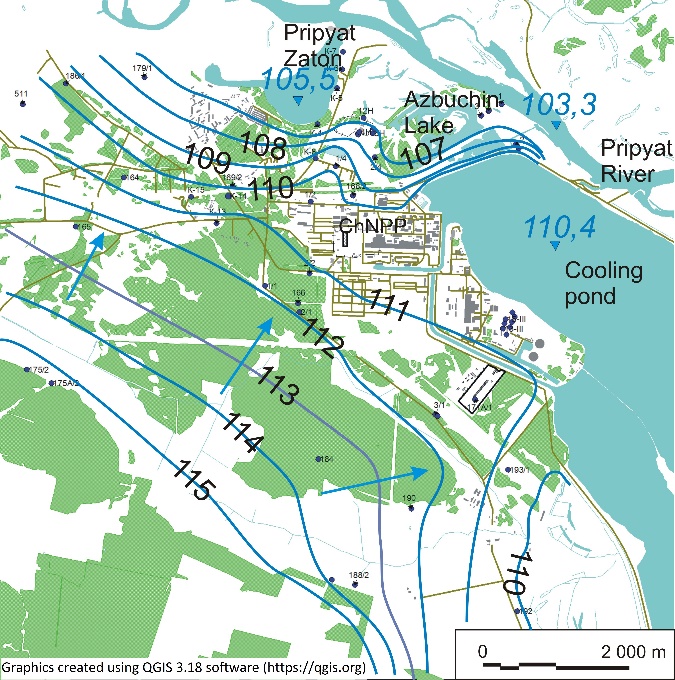 | 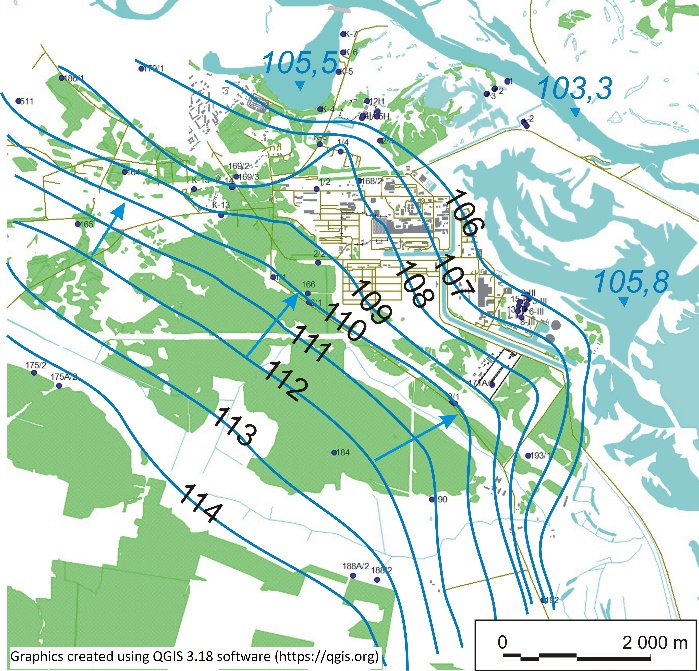 |
| --- | --- |
| (a) Hydraulic head isolines in 2014  (yearly mean values, m a.s.l.) | (b) Hydraulic head isolines in 2019 (m a.s.l.) (yearly mean values, m a.s.l.) |
| 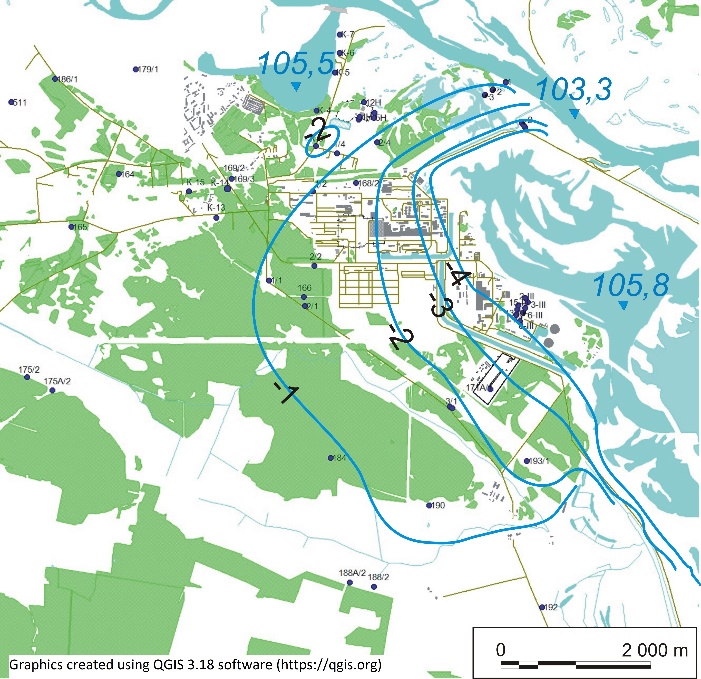 | |
| (c) Decrease of groundwater levels from 2014 to 2019 (m) | |

Figure S1.2. Changes of groundwater levels in the unconfined aquifer in Quaternary deposits at the ChNPP site from 2014 to 2019 due to decommissioning of the cooling pond.

**Confined aquifer in Eocene deposits**

Clay marl deposits of the Kiev suite of Eocene are the first regional aquitard, which separates the unconfined aquifer and confined aquifer in the Eocene sandy deposits. The thickness of the Kiev marl layer ranges from several meters to several tenths of meters, in some locations “hydraulic windows” are present (see Figure 2). The hydraulic conductivity of the marl layer is estimated in the range 2 10^-4^ - 2 10^–2^ m day^-1^, the higher values being more typical for the river floodplain areas. The confined aquifer is composed of marine deposits of Buchakskaya and Kanevskaya suites of Eocene, represented by fine sands with the inter-layers of sandstone, aleurolite and clay. The total thickness of the aquifer varies from 30 to 48 m. Transmissivity of the Eocene aquifer is estimated at 90 – 250 m^2^ day^-1^. The groundwater chemical composition is of hydrocarbonate calcium and calcium-potassium type. The total dissolved solids do not exceed 0.8 mg l^-1^ (Dzhepo and Skalskyy, 2002; Sobotovich and Olshtynsky, 1992). This aquifer in is exploited by Pripyat Town groundwater intake wells, which provide a potable and technological water source for the Chernobyl NPP.

In general, the environmental conditions in the 30-km zone (i.e., humid climate, generally flat landscape with high permeability and low sorption capacity surficial deposits) favor intense migration of fallout radionuclides to the groundwater system.

Changes of hydrogeological conditions at ChNPP site due to decommissioning of the cooling pond

The hydrogeological conditions at the ChNPP site, which were relatively stable during the post Chernobyl accident period, has undergone significant changes due to decommissioning of the cooling pond of the nuclear power plant, which have started in May 2014 (IAEA, 2019). The decommissioning strategy for the pond consisted in lowering of the water level in this reservoir from 110.4 m a.s.l. to 105 – 106 m.a.s.l. (the final levels depend on season and specific location of residual lakes) (see Figure S1.2-a,b). This approach has significant impact on hydrogeological conditions of the surrounding areas including the industrial site of ChNPP, where groundwater levels have decreased by 2-4 m (Figure S1.2-c). The increase of the thickness of the unsaturated zone at the ChNPP site is a favorable factor for improving protection of groundwater from radioactivity sources (contaminated “accident related” soil layers, waste dumps) located in the near-surface soil layers.

# Section 2. Analysis of time trends and additional monitoring data on radionuclide distribution in groundwater in the unconfined aquifer in Quaternary deposits

**2.1 Statistical analysis of trends for time series of ^137^Cs and ^90^Sr activity concentrations in the unconfined aquifer**

This section presents results of Mann-Kendall statistical analysis of trends (Aziz et al., 2003) for groundwater monitoring data in CEZ. Analysis of trends was carried out for following parameters: yearly-averaged ^137^Cs and ^90^Sr activity concentrations in monitoring wells and yearly averaged groundwater levels for the period of 2010 -2019. Calculations used the GSI Mann-Kendall Toolkit software (Connor et al., 2012).

Analysis was carried out for the set of 73 monitoring wells, which were included to the groundwater monitoring program of Ecocenter as of 2018-2019. The wells belonging to monitoring networks of engineered radioactive waste disposal facilities (RWDS) were not included to analysis, as these last facilities are not within the scope of the reported study. Several wells were not included, as these wells have dried up as a consequence of the drainage of the cooling pond.

Results of trend analysis for individual wells are provided in the Annex 5 to Supplemental Information. The analyzed monitoring data set is provided in Annex 6. Based on this analysis, for ^137^Cs only 7 wells (<10%) from 72 analyzed monitoring wells show ‘Increasing’ or ‘Probably increasing’ time trends, while all other wells show non-increasing trends. The trend analysis for activity levels for ^90^Sr shows that 34 wells (47%) demonstrate ‘Decreasing’ or ‘Probably decreasing’ trends, 5 wells (7%) show ‘Increasing’ or ‘Probably increasing’ trends, while for other wells results suggest non-increasing trends (46%). Three from 5 wells with increasing ^90^Sr trends are situated at locations influenced by seepage from contaminated surface water bodies (Pripyatv Zaton and Semikhody Zaton), while 1 well is situated at the waste dump site and one other at location with contaminated topsoil.

Groundwater levels in most monitoring wells (74%) show tendency to decrease in 2010 – 2019 reflecting the consequences of the water level drawdown in the cooling pond, which started in 2014.

**2.2 Graphs of combined data on surface contamination and groundwater contamination by ^137^Cs and ^90^Sr in the unconfined aquifer**

1. Graph for ^137^Cs
2. Graph for ^90^Sr

Figure S2.1. Graphs of combined data on surface contamination and groundwater contamination by ^137^Cs and ^90^Sr in monitoring wells sampling the unconfined aquifer in CEZ (as of 2019). Surface contamination data are based on decay-corrected data of (Kashparov et al., 2003).

**2.3 Vertical distribution of ^137^Cs and ^90^Sr in the unconfined aquifer**

Vertical distribution of ^137^Cs and ^90^Sr in the unconfined aquifer in Quaternary deposits in 2019 is illustrated in Figure S2.1. The ^90^Sr concentration usually shows decrease with depth, which is better pronounced in the areas with higher level of groundwater contamination of the upper part of the aquifer such as waste dump sites (see Figure S2.1-a). The ^137^Cs concentration in groundwater usually shows relatively small variability with the depth, being close to the analytical detection limit for this radionuclide.

(a) Well cluster 2/1, 2/1D (“Stroybaza” waste dump)

(b) Well cluster 185/Q1, Q2, Q3 (site with the topsoil contamination)

Figure S2.2. Vertical distribution of activity of ^137^Cs and ^90^Sr in clusters of multilevel monitoring wells sampling the unconfined aquifer in Quaternary deposits in 2019. Error bars show standard deviation of data during a year (Location of wells is shown at Fig.S10.3).

# Section 3. Detailed studies of groundwater contamination at radioactive waste dump sites

Apart from the monitoring program carried out by “Ecocenter”, groundwater contamination at waste dump sites was studied in several other projects, which are briefly summarized below. The most comprehensive groundwater monitoring studies were carried out within the international radioecological research projects Chernobyl Pilot Site Project (CPS Project, 1999–2004) and Experimental Platform in Chernobyl (EPIC, 2004–2012) (Dewière et al., 2004; Van Meir et al., 2009; Kashparov et al., 2012; Bugai et al., 2012a). These projects studied radionuclide migration from the near-subsurface radioactive waste burial (Trench no.22) at the “Red forest” site. The experimental site near the trench no.22 was equipped with a detailed network of small diameter (1-2 inches) PVC multi-level monitoring wells with 20 cm long screens to the unconfined aquifer in Quaternary deposits (ranging in depth from 3 to 8 m), which allowed the characterization of the ^90^Sr plume in groundwater extending to ~10-15 m downstream from the source and extending 3-4 m in the vertical direction below the groundwater table. Maximum ^90^Sr concentrations in groundwater reaching 3500 Bq l^-1^ were revealed in the upper part of the aquifer (depth interval 1-2 m below groundwater table) immediately downstream (4-6 m distance) of the trench (as of 2001-2003) (Dewière et al., 2004; Van Meir et al., 2009).

A study of ^239,240^Pu and ^238^Pu in groundwater carried at the same site in 2005-2006 by Levchuk et al. (2009) revealed a plutonium plume, which generally correlated with the ^90^Sr plume (in terms of geometry and maximums of activity). Maximum activity of Pu isotopes in groundwater reached ~ 200 mBq l^-1^ for ^239,240^Pu and ~ ~100 mBq l^-1^ for ^238^Pu. In-situ ultrafiltration tests suggested Pu association with a low-molecular organic compounds (< 5 kDa), which were presumably present in groundwater due to degradation of organic waste inside the trench no.22 (Levchuk et al., 2009).

Data on high levels of radionuclides in groundwater in the immediate vicinity (first meters) downstream of waste burials were obtained also in one-time sampling surveys of groundwater with the help of temporary monitoring wells with 0.5 – 1 m long screens installed to the upper part of the unconfined aquifer or using the push-drill auger (Ledenev et al., 1995; Antropov et al., 2001; Bugai et al., 2020a). Results of these studies are summarized in Table S3.1. Maximum concentrations of ^90^Sr measured in in the vicinity of waste burials during different surveys are plotted in Figure S3.1

Table S3.1. Results of one-time groundwater surveys of waste dump sites in the vicinity of the ChNPP carried out in 1992-2015 (data for 1992-1998 are taken from (Antropov et al., 2001); data for 2015 are taken from (Bugai et al., 2020a)).

| Waste dump site * | Waste burial | Year of survey | Maximum radionuclide activity concentration, Bq l^-1^ | | |
| --- | --- | --- | --- | --- | --- |
|  |  |  | ^90^Sr | ^137^Cs | ^239+240^Pu |
| “Red Forest” | 19-T | 1992 | 3600 | no data | 0.42 |
|  | 20-T | 1992 | 30000 | no data | 0.33 |
|  | 29-T | 1992 | 14000 | no data | 0.15 |
|  | 22-T | 1998 | 16000 | 0.3 | 0.18 |
|  | 4-BТ * | 1998 | 2700 | 350 ** | 5.0 |
|  | 1-B | 2015 | 1200 | 0.1 | no data |
|  | 4-BT | 2015 | 3500 | no data | no data |
|  | 6-BT | 2015 | 7100 | no data | no data |
|  | 7-BT | 2015 | 1800 | no data | no data |
|  | 10-T | 2015 | 960 | no data | no data |
|  | 22-T | 2015 | 11000 | 0.22 | no data |
| “Yanov Station” | 14-T | 1995 | 3990 | no data | no data |
|  | B-26 | 2015 | 732 | <0.04 | no data |
| “Neftebaza” | 4-T | 1998 | 613 | 0.4 | 0.039 |
|  | 201-T | 1998 | 274 | 0.3 | <0.001 |
|  | 201-T | 2015 | 3.0 | 0.17 | no data |
|  | 202-T | 2015 | 14.0 | 0.07 | no data |
|  | 204-T | 2015 | 1.4 | 0.6 | no data |
| “Stroybaza” | 33-B | 2015 | 17800 | 0.08 | no data |

Notes: * - Location of different waste dump sites is shown in Figure M1 (Methods);

** High ^137^Cs and ^239+240^Pu values for burial 4-BT can be explained by the fact that the burial at the time of sampling was flooded by groundwater, and sample was taken directly from the water-saturated waste material.

Figure S3.1 The maximum ^90^Sr concentrations in groundwater in the vicinity of waste dump sites measured in the course of one-time surveys in 1992 – 2015. Data points correspond to individual waste burials (based on data of (Antropov et al., 2001; Bugai et al., 2020a).

The highest concentrations of ^90^Sr in groundwater of an order of 1000 – 10000 Bq l^-1^ were observed at “Red forest” and “Stroybaza” waste dumps, which contain higher activity material. These values are 1-2 orders of magnitude higher than ^90^Sr concentrations measured in monitoring wells of “Ecocenter”. This demonstrates the influence of location and design of monitoring wells on results of monitoring observations (it should be noted that monitoring wells used by ‘the Ecocenter” often have 12 m long screens and produce vertically-averaged samples). Data on ^137^Cs in groundwater listed in Table S3.1 are in agreement with the higher end values of ^137^Cs concentrations reported by “Ecocenter”. Data of Table S3.1 on ^239,240^Pu in groundwater are consistent with the data obtained at experimental site near the Trench no.22 discussed earlier. The Figure S3.1 shows a tendency to a decrease of maximum ^90^Sr groundwater concentrations in time from 1992 to 2015. This may be related to natural attenuation process in groundwater, discussed in this article.

# Section 4. Data on groundwater contamination by Pu isotopes and ^241^Am

Table S4.1. Plutonium and americium activity concentrations in groundwater in the CEZ in 2018 (Location of wells is shown at Fig.S10.3).

| Site | Radionuclide activity concentration MEAN (MIN – MAX), Bq m^-3^ | | |
| --- | --- | --- | --- |
|  | ^238^Pu | ^239+240^Pu | ^241^Am |
| Waste dumps  (wells 1/1, 1/2, 2/2, 3/1, K-8) | 0.4  (0.08 – 0.7) | 0.9  (0.2 – 1.6) | 1.9  (0.5 – 3.1) |
| *Seepage from water bodies* |  |  |  |
| Pripyat Zaton  (well K-5) | 0.07 | 0.21 | 0.43 |
| Azbuchin Lake  (wells 1, 1A, 2, 2A, 3, 3A) | 0.03  (0.01 - 0.05) | 0.09  (0.03 – 0.12) | 0.18  (0.08 – 0.23) |

# Section 5. Groundwater contamination caused by seepage from surface water bodies

High levels of groundwater contamination by strontium-90 which show increasing trends in concentration levels were observed in recent period in a number of monitoring wells situated in the vicinity of surface water bodies located in the 10-km zone of ChNPP: the former cooling pond, Pripyat Zaton and Azbuchin Lakes. Example monitoring data for wells K-6 and K-7 situated close to the Pripyat Zaton Lake are shown at Figure S5.1. The Pripyat Zaton Lake represents the dead channel of the Pripyat River separated from the river channel by protective dike. The water level in the Pripyat Zaton (105.5 – 106 m a.s.l.) is 2.5 - 3 m higher than the mean water level in Pripyat River (103.5 m a s.l.). Groundwater contamination in this and other similar locations is likely influenced of seepage of water from the surface water body towards Pripyat River caused by the difference in water pressure head (Figure S5.2). This is evidenced by similarity of hydrochemistry of groundwater in monitoring wells close to Pripyat Zaton to that one of this surface water reservoir (while other unconfined aquifer areas, such as “Red Forest”, are recharged by meteoric waters and have a distinct hydro-chemical “fingerprint”) (Bugai et al., 2020a). The strontium-90 monitoring data reflect passing of leading edges of radionuclide plumes originating from surface water bodies past monitoring stations.

In the course of seepage, the surface water passes through contaminated bottom sediments containing nuclear fuel particles. This can cause additional leaching of radionuclides from FP to pore water, and can result in groundwater concentrations of ^90^Sr, which are higher compared to historic contaminant levels in surface water. In particular, such groundwater contamination patterns were observed in the monitoring wells situated at the dam between the cooling pond and Pripyat River. In different locations of the dam of the cooling pond, maximum ^90^Sr levels in groundwater were observed in 1990 – 1995 and in 1998-2004 (IAEA, 2019). The time dynamics of ^90^Sr concentrations in monitoring wells in different locations was likely determined by variations of seepage rates from contaminated surface water bodies, radionuclide retardation in subsurface due to sorption on aquifer materials, and by initial inventory and leaching behavior of fuel particles in contaminated bottom sediments of reservoirs.

Figure S5.1. ^90^Sr groundwater monitoring data for well3 K-6 and K-7 influenced by seepage from Pripyat Zaton lake. Vertical bars represent standard deviation of data during a year.


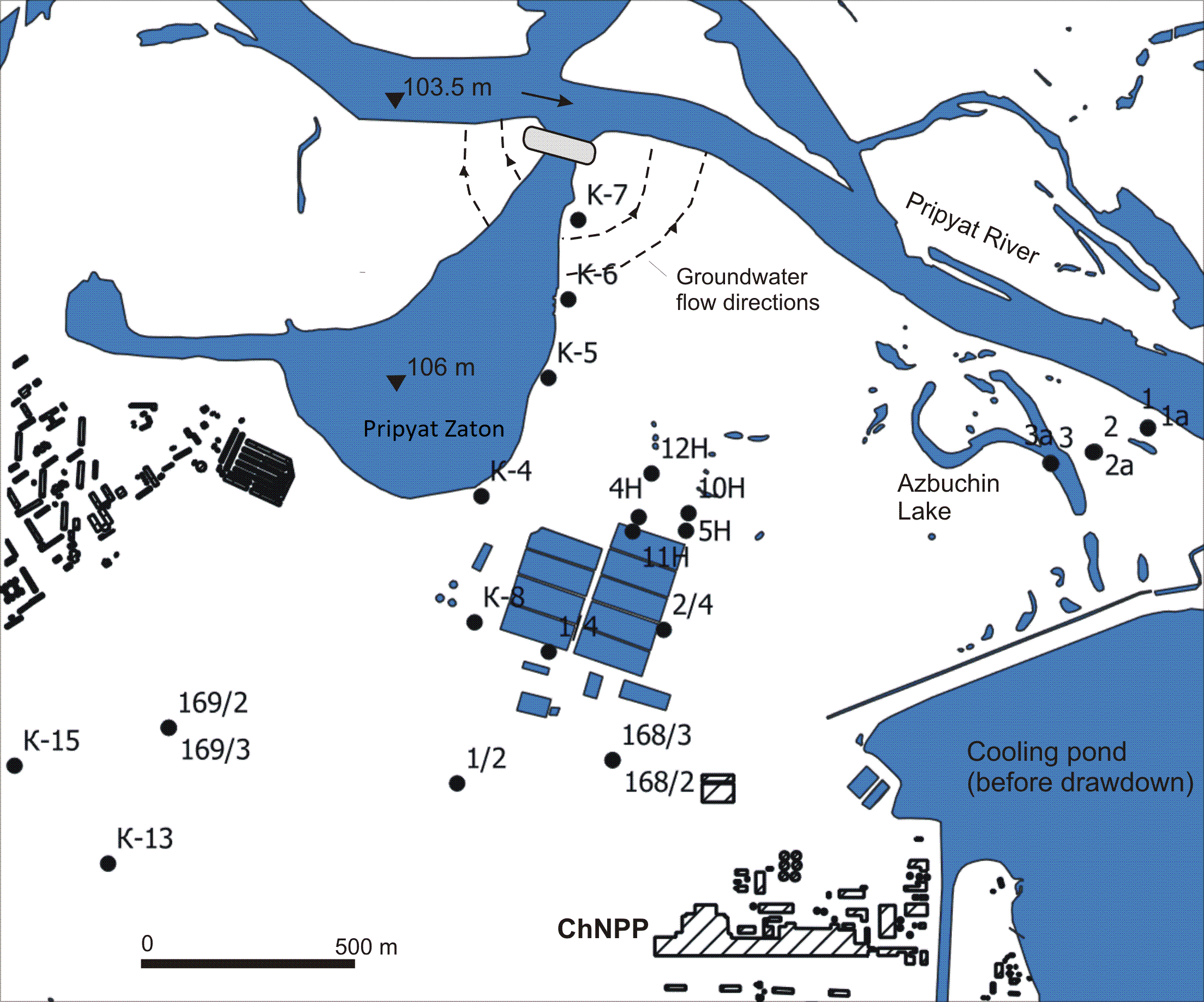


Figure S5.2. Location of monitoring wells and groundwater flow patterns in the vicinity of Pripyat Zaton Lake.

# Section 6. Groundwater monitoring data for deep confined aquifers in CEZ

**6.1 Monitoring data for the confined Eocene aquifer**

|  |
| --- |
| 1. ^137^Cs |
|  |
| 1. ^90^Sr |
| Figure S6.1 Monitoring data for the confined Eocene aquifer. (Vertical bars represent standard deviation of data during a year). |

**6.2 Monitoring data for the confined Cretaceous chalk aquifer**

|  |
| --- |
| 1. ^137^Cs |
|  |
| 1. ^90^Sr |
| Figure S6.2 Monitoring data for the confined Cretaceous chalk aquifer. (Vertical bars represent standard deviation of data during a year). |

# Section 7. Groundwater dating studies and estimation of residence times of groundwater and radioactive contaminant (^90^Sr) in subsurface

The text below briefly reviews groundwater isotope dating and modeling studies aimed at estimation of groundwater and radioactive contaminant (first of all ^90^Sr) residence times from radionuclide sources in the 10-km zone of ChNPP to the surface water bodies and to water wells exploiting the confined aquifer in Eocene deposits.

**Groundwater and radionuclide residence times to surface water system**

The natural tracer (^3^H/^3^He) and anthropogenic tracer (CFC) groundwater dating studies carried out by Le Gal La Salle et al. (2012) for the unconfined aquifer in Quaternary deposits “Red Forest” waste dump site resulted in apparent groundwater ages from 1–3 years in the shallow depth below the groundwater table to 50–60 years at 27 m (bottom depth).

Bugai et al. (2010) used the described above ^3^H / ^3^He dating values to calibrate the MODFLOW-based cross-section groundwater flow model for the system of Quaternary (unconfined) and Eocene (confined) aquifers within the terrace of the Pripyat River where the “Red Forest site” is situated. Results of calculations of groundwater flow in subsurface within the first river terrace (where “Red Forest” waste dumps are situated) towards the Pripyat River are presented in Table S7.1 (column 1 and 2). Groundwater travel times (T_gw_) predicted by the model and the well-known retardation equation were used to get lower bound estimates of ^90^Sr travel times (T_Sr90_) in the subsurface for corresponding fractions of groundwater discharge to Pripyat River (Table S7.1, column 3):

T_Sr90_ = T_gw_ × R, R = 1 + ρ/m × Kd,

Where R is retardation factor, ρ is sediment bulk density (1.6 kg/dm^3^), m is sediment porosity (0.3) and Kd is sorption distribution coefficient. The above calcualtion employed the lower end Kd value of 2 l kg^-1^ of ^90^Sr for sandy deposits in CEZ (Bugai et al., 2020b).

Groundwater travel time estimates from the major waste dump sites and Sarcophagus to surface water bodies in the 10-km zone of ChNPP derived in Buckley et al. (2002) using the regional groundwater flow model of the 30-km zone of ChNPP are presented in Table S7.2 (columns 1-3). We used this information to derive lower bound estimates of ^90^Sr travel times in the subsurface (Table S7.2, column 4). The procedure is similar to the one used in Table S7.1.

Table S7.1 Calculated distribution of ages of groundwater discharging to the Pripyat River and associated ^90^Sr travel times in the subsurface for the groundwater system encompassing the “Red Forest” site in CEZ using groundwater flow model described in (Bugai et al., 2010).

| **Fraction of groundwater discharge to Pripyat River, %** | **Estimated groundwater age, years *** | **Conservatively estimated ^90^Sr residence time, years **** |
| --- | --- | --- |
| 1 | 2 | 3 |
| 15% | < 20 | < 200 |
| 15 – 30% | 20 – 50 | 200 – 500 |
| 30 – 65% | 50 – 130 | 500 – 1300 |
| 65% - 100% | 130 – 500 | 1300 – 5000 |

Notes: * - travel time estimates do not include time of transport in the unsaturated zone; ** - ^90^Sr residence time calculations assume conservative Kd=2 l kg^-1^ (retardation factor R~10)

Table S7.2. Groundwater and radionuclide travel times in subsurface from waste dump sites and Sarcophagus (Buckley et al., 2005)

| **Radioactively contaminated site** | **Groundwater discharge contour** | **Estimated groundwater travel time, years*** | **Estimated ^90^Sr travel time, years**** |
| --- | --- | --- | --- |
| 1 | 2 | 3 | 4 |
| “Red Forest” waste dump | Pripyat River | 290 | 2900 |
| “Yanov station” waste dump | Pripyat Zaton | 55 | 550 |
| “Stroybaza” waste dump | Pripyat Zaton | 60 | 600 |
| “Sarcophagus” | Residual lake at cooling pond site | 45 | 450 |

Notes: * - travel time estimates do not include time of transport in the unsaturated zone; ** - ^90^Sr residence time calculations assume conservative Kd=2 l kg^-1^ (retardation factor R~10)

**Groundwater and radionuclide residence times to water wells exploiting the Eocene aquifer**

Although there is no known groundwater date from the deep aquifers in Chernobyl, given the geology and extent of the aquitard layers, it is expected that the residence time of the groundwater is large, as has been seen in hydraulically similar aquifer systems elsewhere in the world (Habermehl 2020; Abouelmagd et al., 2014).

Calculations of travel times of groundwater to the water well exploiting the confined Eocene aquifer at ChNPP site using a numerical 2D radial flow model was carried out by Bugai et al. (1996). The modeling predictions of groundwater travel times are consistent with tritium sampling data for these wells likely reflecting the “bomb tritium” pulse from nuclear tests in the 1960’s.

Results of calculations of groundwater and ^90^Sr travel times to water wells exploiting the confined aquifer in Eocene deposits are summarized in Table S7.3

The presented modeling results (Tables S7.1, S7.2) suggest that a relatively small fraction of subsurface flow at groundwater discharge contours (water supply wells, rivers) is composed of “younger” waters that can potentially carry radioactivity.

The modeling results (Tables S7.2, S7.3) also show long enough residence time of ^90^Sr, which is retarded by sorption, from the sources of contamination to the river network and water wells exploiting the Eocene aquifer, to allow ^90^Sr to decay to safe levels.

Table S7.3. Calculated distribution of ages of groundwater extracted by water well exploiting confined aquifer in Eocene deposits at ChNPP site and associated ^90^Sr travel times in the subsurface using groundwater flow model described in (Bugai et al., 1996).

| **Fraction of groundwater discharge, %** | **Estimated groundwater age, years *** | **Conservatively estimated ^90^Sr residence time, years **** |
| --- | --- | --- |
| 5 | 12 – 20 | 120 – 200 |
| 5-50 | 20 – 50 | 200 – 500 |
| 50 - 100 | 50 – 600 | 500 – 6000 |

Notes: * - travel time estimates do not include time of transport in the unsaturated zone; ** - ^90^Sr residence time calculations assume conservative Kd=2 l kg^-1^ (retardation factor R~10)

# Section 8. Groundwater monitoring data for water supply wells used by ‘samosely’ in the Chernobyl exclusion zone

Table S8.1. Volumetric activity of radionuclides in potable water from dug wells and boreholes of “samosely” (self-settlers) in CEZ in 2019. WHO Drinking water limit is 10 000 Bq m^-3^ for both radionuclides. Value in parentheses is the analytical measurement error (%).

| **Sampling site** | **Number of well / borehole** | **^137^Сs, Bq/m^3^** | **^90^Sr, Bq/m^3^** |
| --- | --- | --- | --- |
| 1 | 2 | 3 | 4 |
|  | | | |
| **Permissible activity in drinking water according to DR-2006** | | **2** | **2** |
|  | | | |
| **Lubyanka Village** | | | |
| 1 Travnaya Str., 50 | Dug well | 50 (10) | 2100 (10) |
| **Kupovate Villag** | | | |
| Stepova Str, 10 | Dug well | 0.7 (30) | 19 (19) |
| Sagirna Str, 10 | Dug well №17 | 5 (21) | 10 (31) |
| Kovpaka Str, 20 | Dug well № 3 | 6 (12) | 170 (15) |
| Partizanska Str, 2 | Dug well № 10 | 2 (30) | 140 (17) |
| **Opachichy Village** | | | |
| Sampling location 1  Sampling location 2 | Dug well  Dug well № 15 | 47 (5)  9 (30) | 41 (17)  200 (17) |
| **Otashiv Village** | | | |
| Sampling location 1 | Dug well | 4 (30) | 47(11) |
| **Teremtsy Village** | | | |
| Sampling location 1  Sampling location 2 | Dug well № 11  Dug well | 4 (18)  <MDA* | 4 (74)  7 (41) |
| Sampling location 3 | Dug well | <MDA | 5 (10) |
| Sampling location 4 | Dug well | 7 (10) | 21 (19) |
| **Paryshiv Village** | | | |
| Sampling location 1 | Dug well | 8 (19) | 62 (11) |
| **Chernobyl Town** | | | |
| Livarny Str, 14 | Dug well | 110 (7) | 110 (10) |
| Livarny Str, 13 | Dug well | <MDA | 61 (15) |
| Livarny Str, 16 | Dug well | 10 (12) | 210 (14) |
| Paroplavny Str. | Borehole | 64 (30) | 120 (11) |

Note: MDA – minimum detected activity (see “Methods” section for detail)

# Section 9. Data on ^90^Sr and ^137^Cs concentrations in the Pripyat River water

(a)

(b)

Figure S9.1. Yearly averaged ^137^Cs (a) and ^90^Sr (b) activity concentrations in water of Pripyat River measured near Chernobyl Town and respective exponential trends (doted lines). (Vertical bars represent standard deviation of data during a year).

# Section 10. Supplemental maps and tables

**Information on landscape polygons**


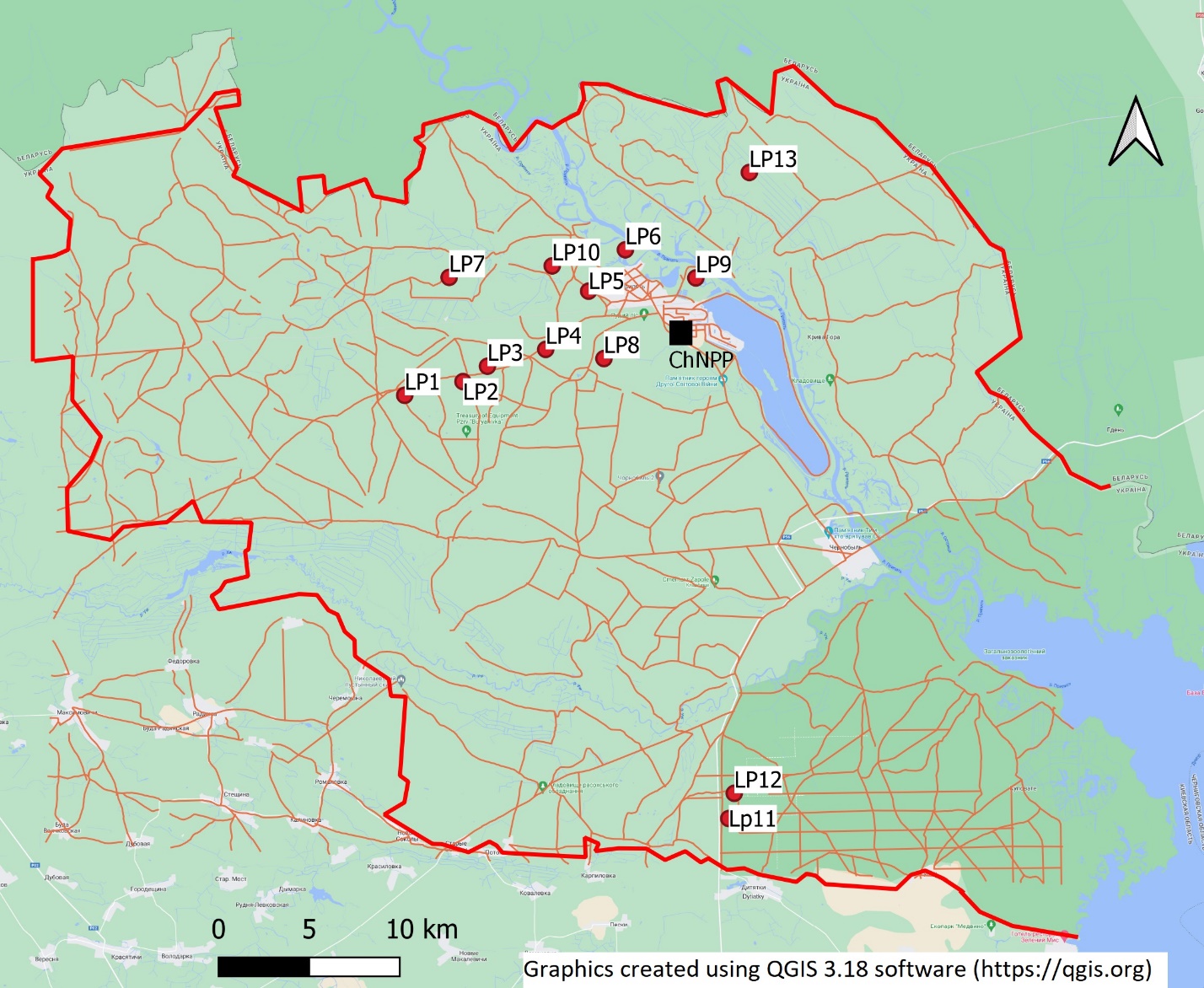


Figure S10.1. Map showing location of location of polygons for soil sampling in the Chernobyl exclusion zone.

Table S10.1. Characteristics of landscape polygons in CEZ.

| Number of landscape polygon | Geographical coordinates | | Soil contamination density in 0-20 cm layer, kBq/m^2^ (as of 2021) | | Landscape (geomorphology) | Soil type | Vegetation cover |
| --- | --- | --- | --- | --- | --- | --- | --- |
|  | Latitude | Longitude | ^137^Cs | ^90^Sr |  |  |  |
| 1 | 51,353763 | 29,900001 | 690 | 54 | Glacial plateau | Sod-podzols, clayey-sandy | Forest |
| 2 | 51,357926 | 29,922222 | 730 | 98 | Glacial plateau | Sod-podzols, clayey-sandy | Forest |
| 3 | 51,365704 | 29,940000 | 1180 | 240 | Glacial plateau | Sod-podzols, clayey-sandy | Forest |
| 4 | 51,374593 | 29,986389 | 1570 | 180 | Glacial plateau | Sod-podzols, clayey-sandy | Forest |
| 5 | 51,402926 | 30,024444 | 1280 | 250 | Terrace of Pripyat River | Sod-podzols clayey-sandy; in depressions - – peaty-clayey | Forest – 40%; meadow – 60% |
| 6 | 51,423482 | 30,039722 | 2550 | 480 | Floodplain of Pripyat River | Sod-podzols, sandy | Forest – 20%; meadow – 80% |
| 7 | 51,410704 | 29,913333 | 300 | 49 | Terrace of Pripyat River | In pine forest – sod-podzols, clayey-sandy; in birch forest – sod-podzols, peaty-clayey | Forest – 70%, meadow – 30% |
| 8 | 51,370071 | 30,033751 | 3610 | 910 | Terrace of Pripyat River | Sod-podzols, clayey-sandy | Forest -60%; meadow - 40% |
| 9 | 51,410489 | 30,101936 | 12020 | 2280 | Floodplain of Pripyat River | In the oak forest -sod-pozols, clayey, in the meadow - transitional peat-bog | Forest – 25%; meadow – 55%; wetland – 20% |
| 10 | 51,418193 | 29,994011 | 334 | 49 | Terrace of Pripyat River | Sod-podzols, sandy | Forest – 40%, meadow – 60% |
| 11 | 51,143001 | 30,130241 | 73 | 10 | Glacial plateau | Sod-podzols, clayey-sandy | Forest |
| 12 | 51,155998 | 30,132199 | 108 | 33 | Glacial plateau | Peaty-clayey | Forest |
| 13 | 51,465355 | 30,152298 | 3157 | 126 | Glacial plateau | Sod-podzols, clayey-sandy | Forest |


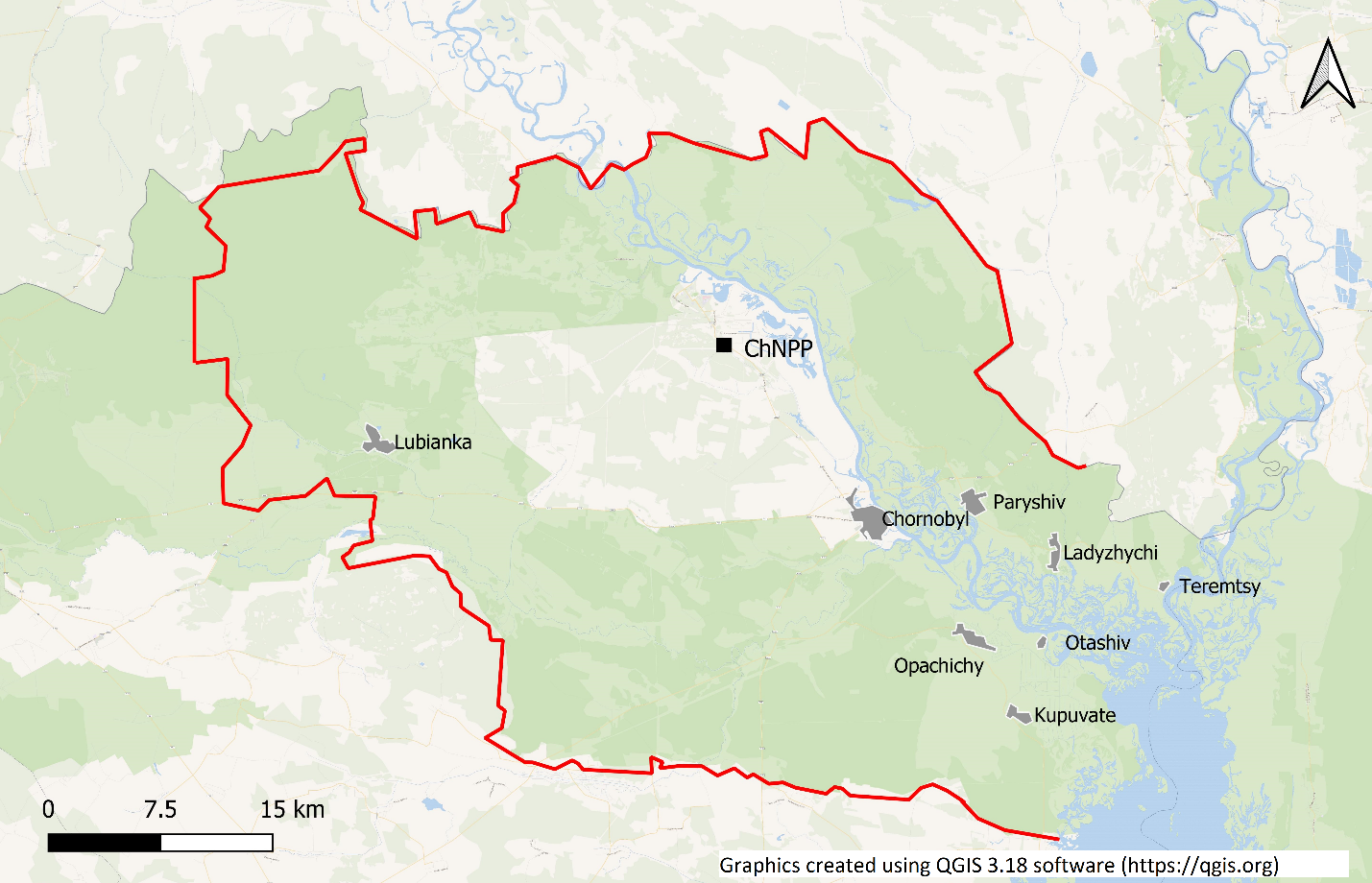


Figure S10.2. Map showing location of villages with ‘samosely’ in the Chernobyl exclusion zone.


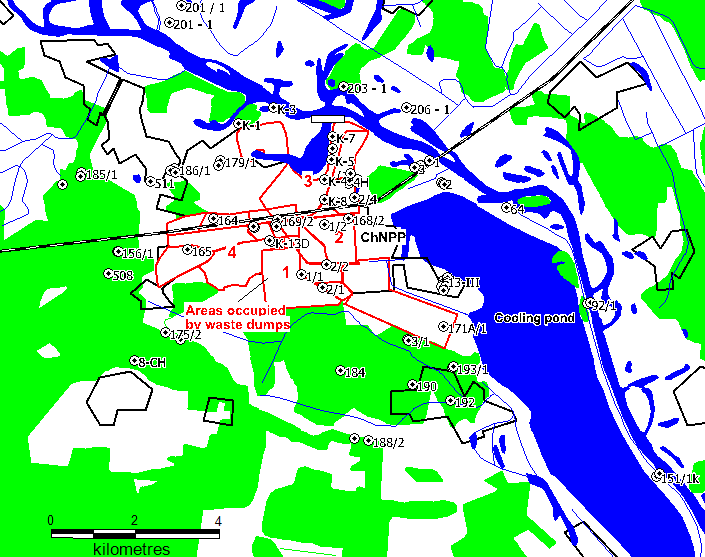


Figure S10.3. Layout of groundwater monitoring well network of the SSE “Ecocenter” in the 10-km zone of ChNPP (the contour of the cooling pond is shown as of 2014). Notation of waste dumps: 1 – “Red forest”, 2 – “Stroybaza”, 3 – “Neftebaza”, 4 – “Yanov station”.

# Supplemental References

Abouelmagd A., Sultan M., Sturchio N., Soliman F., Rashed M., Ahmed M., Chouinard K. (2014). Paleoclimate record in the Nubian Sandstone Aquifer, Sinai Peninsula, Egypt. Quaternary Research. 81(1), 158-167. <https://doi.org/10.1016/j.yqres.2013.10.017>

Antropov V. M., Bugai D., Dutton L.M.C., Gerchikov M., Kennett E. J., Ledenev A. I., Novikov A. A., Rudko V., Ziegenhagen J. (2001). Review and analysis of solid long-lived and high level radioactive waste arising at the Chernobyl Nuclear Power Plant and the restricted zone, EUR 198197 EN. NNC Ltd, Manchester. <https://doi.org/10.13140/RG.2.2.31635.17442/1>

Aziz J. J., Ling. M., Rifai, H. S., Newell C. J., & Gonzales J. R. (2003). MAROS: A Decision Support System for Optimizing Monitoring Plans. Ground Water, 41(3), 355–367. <https://doi.org/10.1111/j.1745-6584.2003.tb02605.x>

Bondarkov M., Oskolkov B., Gashchak S., Kireev S., Maksimenko A., Proskura N., Jannik G., Farfán E. (2011). Environmental radiation monitoring in the Chernobyl Exclusion Zone-history and results 25 years after. Health Phys. 101 (4), 442-485. <https://doi.org/10.1097/HP.0b013e318229df28> .

Buckley M.J., Bugai D., Dutton L.M.C.D., Gerchikov M.Y., Kashparov V.A., Ledenev A., Voitzehovich O., Weiss D., Zheleznyak M. (2002) Drawing Up and Evaluating Remediation Strategies for the Chernobyl Cooling Pond. Final Report, Rep. C6476/TR/001/2002, NNC, Knutsford, UK, 2002. - 92 p.

Bugai D.A., Fourre E., Jean-Baptiste P., Dapoigny A., Baumier D., C. Le Gal La Salle, Lancelot J., Skalskyy A.S., Van Meir N. (2010) Estimation of groundwater exchange at Chernobyl site using the data of the isotope dating and hydrogeological modeling. Geological Journal (Ukraine). (4), 119-124 (in Ukrainian)

Bugai D., Skalskyy A., Dzhepo S., Kubko Yu., Kashparov V., Van Meir N., Stammose D., Simonucci C., Martin-Garin A. (2012a). Radionuclide migration at experimental polygon at Red Forest waste site in Chernobyl zone. Part 2: Hydrogeological characterization and groundwater transport modeling. Applied Geochemistry. 27 ( 7), 1359–1374. <https://doi.org/10.1016/j.apgeochem.2011.09.028>.

Bugai D., Bayer P., Haneke K., Sizov A., Tretyak O., Kubko Yu., Kireev S., Molitor N. (2020a). Radioactive contamination of groundwater at waste dump sites in Chernobyl Exclusion Zone. Geological Journa (Ukraine)l, No. 2 (371), 27—38. [https://doi.org/10.30836/igs.1025-6814. 2020.1.196974](https://doi.org/10.30836/igs.1025-6814.%202020.1.196974)

Bugai D., Smith, J., Hoqu, M.A. (2020b) Solid-liquid distribution coefficients (Kd-s) of geological deposits at the Chernobyl Nuclear Power Plant site with respect to Sr, Cs and Pu radionuclides: A short review (2020b). Chemosphere, Volume 242, 125175 <https://doi.org/10.1016/J.CHEMOSPHERE.2019.125175>

Connor J.A., Farhat S.K., M. Vanderford (2012) Software User’s Manual GSI Mann-Kendall Toolkit For Constituent Trend Analysis/Ver. 1. GSI Environmental Inc., Houston, Texas, 19 p.

Davydchuk V.S. (1996) Physical-geographic conditions and landscape-geochemical peculiarities. In: Baryakhtar V.G. (Ed.), Chernobyl Catastrophe. Naukova Dumka, Kiev, p.185-195. (in Ukrainian)

Dewière L., Bugai D., Grenier C., Kashparov V., Ahamdach N. (2004) ^90^Sr migration to the geo-sphere from a waste burial in the Chernobyl exclusion zone. J.Environ. Radioact. 74, 139–150. <https://doi.org/10.1016/j.jenvrad.2004.01.019>

Dzhepo S., Skalskyy A. (2002) Radioactive contamination of groundwater within the Chernobyl Exclusion Zone, in: Shestopalov, V. (Ed.), Chernobyl disaster and groundwater. A.A. Balkema Publishers, pp. 25–70.

Galuschenko N.G. (1991) Estimate of the seasonal and annual runoff of the rivers of the Pripyat River basin. Trudi UKRNIGMI (Proceedings of the Ukrainian Institute of Hydrology and Meteorology). 240, 1-168 (in Russian).

Habermehl M.A. (2020) Review: The evolving understanding of the Great Artesian Basin (Australia), from discovery to current hydrogeological interpretations. Hydrogeol. J., 28, 13–36. <https://doi.org/10.1007/s10040-019-02036-6>

Kuznetcov V.A. (1973). Geochemistry of the alluvial lithogenesis, Nauka i technika Publishers, Minsk. (in Russian)

Le Gal La Salle C., Aquilina L., Fourre E., Jean-Baptiste P., Michelot J.-L., Roux C., Bugai D., Labasque T., Simonucci C., Van Meir N., Noret A., Bassot S., Dapoigny A., Baumier D., Verdoux P., Stammose D., Lancelot J. (2012) Groundwater residence time downgradient of Trench No. 22 at the Chernobyl Pilot Site: Constraints on hydrogeological aquifer functioning, Applied Geochemistry, 27, 1304-1319, <https://doi.org/10.1016/j.apgeochem.2011.12.006>.

Ledenev A.I., Ovcharov P.A., Mishunina I.B., Antropov V.M. (1995) Results of comprehensive studies of the radiation conditions of temporary localization points for radioactive waste in the exclusion zone of ChNPP. Problems of Chernobyl Exclusion Zone, Issue 2, 46-50. (in Russian)

Levchuk S., Yoschenko V., Kashparov V., Van Meir N., Ardois C., Bugaï D. (2009). Phenomenon of a fast migration of plutonium radioisotops in ground water: Colloids or soluble form? in: Oughton, D.H., Kashparov, V. (Eds.), Radioactive Particles in the Environment. Springer Science+Business Media B.V, pp.157-167. <https://link.springer.com/chapter/10.1007/978-90-481-2949-2_9>

Matoshko A., Bugai D., Dewiere L., Skalskyy A. (2004) Sedimentological study of the Chernobyl NPP site to schematize radionuclide migration conditions. Environm. Geol. 46, 820-830. <https://doi.org/10.1007/s00254-004-1067-3>

Olkhovik Yu.A., Koromyslychenko T.I., Gorgotskaya L.I., Sobotovich E.V. (1992). Estimation of sorption ability of sandy soils in the Near Zone of Chernobyl nuclear power plant. Reports of the Ukrainian Academy of Sciences. 7, 167-171 (in Ukrainian)

Skalskyy A.S., Kubko Y.I. (2000) Filtration models of the Chernobyl NPP site. In: Shestopalov V (ed) Water Exchange and Chernobyl Accident. Vol.2. Modeling of water exchange and radionuclide migration in hydrogeology structures. Ukrainian National Academy of Sciences, Kiev, pp 448-481 (in Russian)

Van Meir N., Bugaï D., Kashparov V. (2009). The Experimental Platform in Chernobyl: An international research polygon in the exclusion Zone for soil and groundwater contamination, in: Oughton, D.H., Kashparov, V. (Eds.), Radioactive Particles in the Environment. Springer Science+Business Media B.V., pp.197-208. <https://link.springer.com/chapter/10.1007/978-90-481-2949-2_13>
